# Role of geographic risk factors and social determinants of health in COVID-19 epidemiology: Longitudinal geospatial analysis in a midwest rural region

**DOI:** 10.1017/cts.2021.885

**Published:** 2021-12-27

**Authors:** Philip H. Wheeler, Christi A. Patten, Chung-Il Wi, Joshua T. Bublitz, Euijung Ryu, Elizabeth H. Ristagno, Young J. Juhn

**Affiliations:** 1 Department of Pediatric and Adolescent Medicine, Mayo Clinic, Rochester, MN, USA; 2 Department of Quantitative Health Sciences, Mayo Clinic, Rochester, MN, USA

**Keywords:** SARS-CoV-2, COVID-19, GIS, risk, outcomes, public health, socioeconomic status, social determinants of health, epidemiology, cohort study

## Abstract

**Background:**

Studies examining the role of geographic factors in coronavirus disease-2019 (COVID-19) epidemiology among rural populations are lacking.

**Methods:**

Our study is a population-based longitudinal study based on rural residents in four southeast Minnesota counties from March through October 2020. We used a kernel density estimation approach to identify hotspots for COVID-19 cases. Temporal trends of cases and testing were examined by generating a series of hotspot maps during the study period. Household/individual-level socioeconomic status (SES) was measured using the HOUSES index and examined for association between identified hotspots and SES.

**Results:**

During the study period, 24,243 of 90,975 residents (26.6%) were tested for COVID-19 at least once; 1498 (6.2%) of these tested positive. Compared to other rural residents, hotspot residents were overall younger (median age: 40.5 vs 43.2), more likely to be minorities (10.7% vs 9.7%), and of higher SES (lowest HOUSES [SES] quadrant: 14.6% vs 18.7%). Hotspots accounted for 30.1% of cases (14.5% of population) for rural cities and 60.8% of cases (27.1% of population) for townships. Lower SES and minority households were primarily affected early in the pandemic and higher SES and non-minority households affected later.

**Conclusion:**

In rural areas of these four counties in Minnesota, geographic factors (hotspots) play a significant role in the overall burden of COVID-19 with associated racial/ethnic and SES disparities, of which pattern differed by the timing of the pandemic (earlier in pandemic vs later). The study results could more precisely guide community outreach efforts (e.g., public health education, testing/tracing, and vaccine roll out) to those residing in hotspots.

## Introduction

The rapid spread of coronavirus disease-2019 (COVID-19) has created a worldwide pandemic with high morbidity and mortality rates [1]. Nationally, there is a disproportionate impact on rural populations in terms of deaths and hospitalizations [2–5]. While rural areas initially experienced lower testing and cases [6,7], since August 2020, the trends have reversed with COVID-19 cases per capita in small/medium metro and non-metro areas exceeding large metro central and fringe areas after mid-August 2020 [8]. Death rates in non-metro areas also exceeded death rates in metro areas from late August to mid-December 2020 [9]. Compared with urban residents, people living in rural areas report less willingness to be vaccinated for COVID-19 [10,11], and, as our community-based survey indicated, less engagement in COVID-19 preventive behaviors, for example, masking [12].

Minnesota’s Governor issued a Shelter-in-Place order from March 27, 2020, to May 13, 2020. The first COVID-19 case in rural parts of the four-county Southeast Minnesota study area (Dodge, Goodhue, Olmsted, and Wabasha Counties) was reported on March 17, 2020. From March through October, the total number of COVID-19 cases was 142,311 in Minnesota and 4880 in the four-county area that is the focus of this study [13], with rural areas accounting for an estimated 41% of area cases based on Rochester Epidemiology Project data (REP; an NIH-funded data linkage system for study populations).

We performed geospatial and temporal trend analysis of COVID-19 experience in rural parts of four Southeast Minnesota counties (50% rural, i.e., with RUCA Code other than 1.1) [14], to examine the influence of geographic factors in COVID-19 epidemiology in a Midwest region. We also examined whether identified hotspots are associated with social determinants of health (SDOH), focusing on socioeconomic status (SES) and housing characteristics. Understanding the effects of where people live within counties, as well as SDOH, could more precisely guide outreach efforts and public health interventions (e.g., COVID-19 testing and vaccination) for rural populations.

## Methods

### Study Setting

Medical records-based research of the area population was performed through access to COVID-19 laboratory test data from the REP database. The REP database includes a majority of residents in the study area, with their inpatient and outpatient clinical diagnoses and address information [5]. Comparing REP data to population estimates from the Census 2018 5-year ACS data, geocoded REP records for those with research authorization covered 75.0% of rural residents in the four-county study area (90,975 of 121,241), ranging from 63.7% in Goodhue County to 93.2% in Olmsted County [5]. Since REP has an overall research authorization level of 90.1% for a 27-county area of which the study area is a part [15] the variation in coverage is the result of residents getting health care from providers not covered by REP.

Similar coverage applies to residents with COVID-positive tests in the region (including both urban and rural parts), with geocoded REP records representing 76.4% of cases (3728 of 4880 cases) through the study period. The rural population of these four counties is 95.3% White (93.3% non-Hispanic White [NHW]), with 3.1% Hispanic of any race, 0.7% Black, 0.5% American Indian, 0.9% Asian, and 2.6% Other/Mixed [14]. Rural portions of the region had a lower proportion of households in poverty (3843 households of 48,401, 7.9%) as compared to urban households (9.6%) [14].

### Study Design and Cohort

This is a population-based retrospective cohort study assessing the temporal (semi-monthly) and geospatial distribution of test-confirmed COVID-19 cases in the rural population from March 17, 2020, to October 31, 2020. We used the geocoded portion of the REP population living in rural areas (see rural classification below) (denominator N = 90,975) and utilized the REP database to identify people who had COVID-19 tests and corresponding test results. For people tested multiple times, the date of the first negative test was retained for temporal analysis purposes, unless superseded by a positive test. In that case, the date of the first positive test was used for temporal analysis. The unit of analysis is thus persons tested (n = 24,243), and not tests. SARS-CoV-2 testing was performed according to manufacturer’s instructions for the real-time reverse transcription polymerase chain reaction (RT-PCR)-based cobas SARS-CoV-2 assay (Roche Molecular Systems, Inc., Branchburg, NJ), which received emergency use authorization from the US Food and Drug Administration. This assay detects the SARS-CoV-2 ORF1ab and E gene sequences; test results were reported as target detected, target not detected, presumptive positive (when only the E gene sequence was detected), or inconclusive (when PCR inhibition was present).

The study was approved by the Mayo Clinic and Olmsted Medical Center Institutional Review Boards.

### Rural Classification

We identified “urban” as populations residing inside the City of Rochester or the block groups (BGs) identified in RUCA Class 1.1 (see map in supplement). All other areas, including smaller cities and townships, were considered rural and included in the analysis [16,17]. By this two-way classification, the four-county region is 50.4% rural and 49.6% urban [14]. The rural population of the 66 townships (unincorporated jurisdictions) in the study area ranged from 170 to 2873 [14]. Township rural REP population density ranged from 4.0 to 115.0 (mean 14.5) per square mile. The population of the 31 rural cities (incorporated jurisdictions) in the study ranged from 91 to 16,338 (median 1268). There are 11 cities with populations over 2500 (urban clusters by Census definition) [18]. City REP population density averaged 553 per square mile.

### Geospatial Analysis

1. Geocoding: The addresses of persons in the REP were geocoded using parcel-based geocoding methods, yielding precise household location and housing characteristics (e.g., apartment, mobile home community [MHC], or single-family house), in relation to the epidemiology of COVID-19.
2. Weighting: Case density was weighted as in other related studies [15]. As positivity of COVID-19 testing depends in part on the level of testing, we accounted for the proportion of persons tested for COVID-19 within the Census block group compared to the overall rural population in each county, by applying a weight derived by the formula: W=(BGpop/Rurpop)/(BGTP/RurTP), where W is the weight, BGpop is the Census block group population, Rurpop is the rural county population, BGTP is the number of tested persons in the Census block group, and RurTP is the number of tested persons in the rural portion of the county. The resulting weights were then applied to each positive test in subsequent analysis steps.
3. Trend analysis: To examine temporal trends in the spatial locations of hotspots, we collected data for COVID-19 cases and testing for all of March 2020 (3/17–3/31), early April (4/1–4/15), late April (4/16–4/30), early May, late May, and so on, mapping concentrations of cases for each time period. For purposes of analysis, due to low numbers of rural cases, we grouped periods March–June (148 city cases, 63 township cases), July–August (194, 129), September (194, 130), October 1–15 (130, 98), and October 16–31 (264, 147).
4. Determining hotspots: We applied a similar geospatial analysis approach as used for our previous studies [19,20]. *For areas within cities,* we mapped the kernel density of weighted positive cases using a half-mile bandwidth. The half-mile bandwidth made it possible to detect the influence of individual apartment complexes, mobile home parks, individual subdivisions, and so on. Larger bandwidths made it harder to detect the influence of these geographic characteristics. Smaller bandwidths increased the number of areas having high weighted case density but lacking cases, due to the influence of positive cases located surrounding but outside the “hotspot.” For each period and for the combined analysis for March through October 2020, we defined “hotspots” as areas with case density in the 90^th^ percentile or higher across rural cities in the four-county area AND with relative difference at least 33% higher than expected case density. Relative difference was derived using the formula RD = (wOCD-ECD)/ECD, where RD is the relative difference, wOCD is the weighted observed case density, and ECD is the expected case density based on average incidence applied to the REP population.

In all but 2 of 66 townships, population density is so low throughout the township that expected case density per square mile is less than 1, such that the relative difference approach described above yielded distorted results. As an alternative, we identified households with positive cases and applied kernel density methods with a one-mile bandwidth to identify positive-case households that were within one mile of and within the same grouped time period as another household with a positive case. We assigned township hotspot status to these concentrations.

### Other Pertinent Variables

Basic demographic characteristics (age, sex, race/ethnicity) were extracted from medical records available from REP. For SES, we used the HOUSES index, a validated individual-level socioeconomic measure linked with addresses at the time of testing (or at the end of study period for those not tested) [21]. Since its original validation, HOUSES index has been widely used for clinical and epidemiological studies concerning 38 different health outcomes and behaviors as well as health care delivery in both children and adults [22–46].

### Data Analysis

Apart from geospatial and temporal trend analysis for COVID-19 cases in the community, we compared sociodemographic characteristics of study subjects within hotspots with those outside hotspots using logistic regression models. Separate analysis was performed for small cities and townships. We also described patterns of COVID-19 laboratory testing and positivity during the study period, stratified by hotspot status (within vs outside hotspots) and locations (small cities vs townships). Geospatial analysis was performed using ArcMap 10.4.1 (produced by ESRI).

## Results

### Characteristics of Study Subjects

Of 90,975 rural residents included in the analysis, 51.7% were female, 92.9% were White (90.1% NHW), 1.0% African American, 0.8% Asian, 0.4% American Indian, and 3.6% other race or two or more races (Other/Mixed); 4.7% reported Hispanic ethnicity. The median age was 42.7 years old (inter-quartile range 21.1–62.1).

### Prevalence of COVID-19, Temporal Trends and Characteristics COVID-19 Cases

**Fig. 1.**
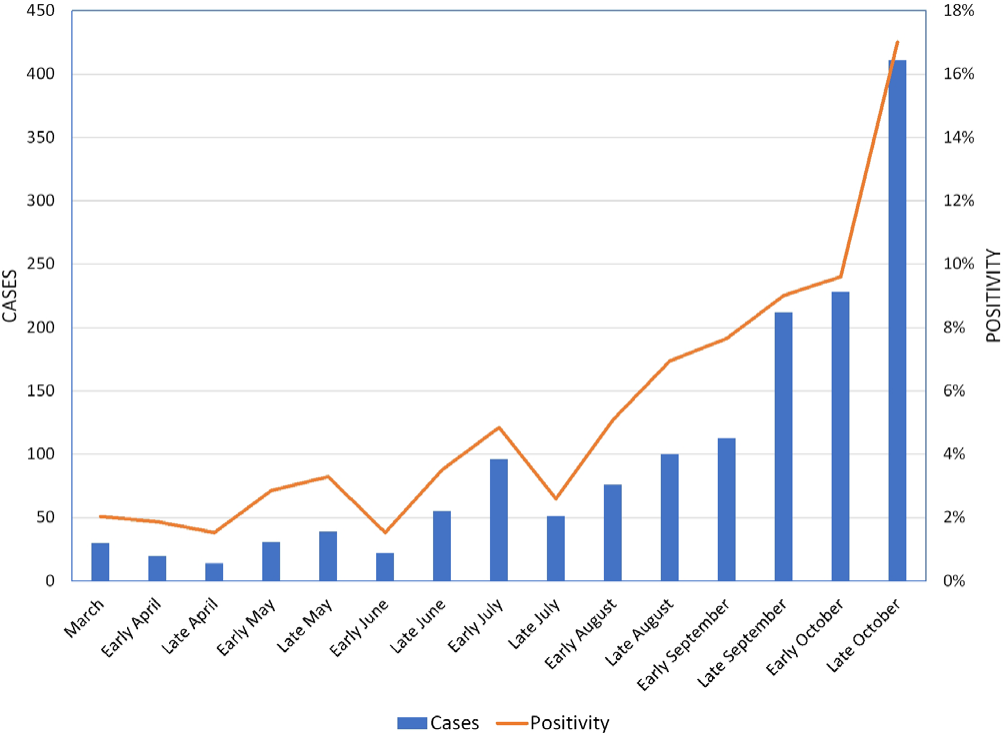
Cases and Positivity by Period COVID-19 Four-County Rural Analysis Southeast Minnesota 3/11–10/31/2020.

A total of 24,243 geocoded rural subjects (26.6%) were tested at least once of whom 1498 (6.2% of tested and 1.6% of rural population) tested positive. Since the first COVID-19 case was confirmed on 3/17/2020, new cases per month peaked in early July, declined in late July and early August, and increased again to a higher peak (four times the July peak) in late October (see Fig. 1). Similar trends were observed for positivity rate.

**Fig. 2.**
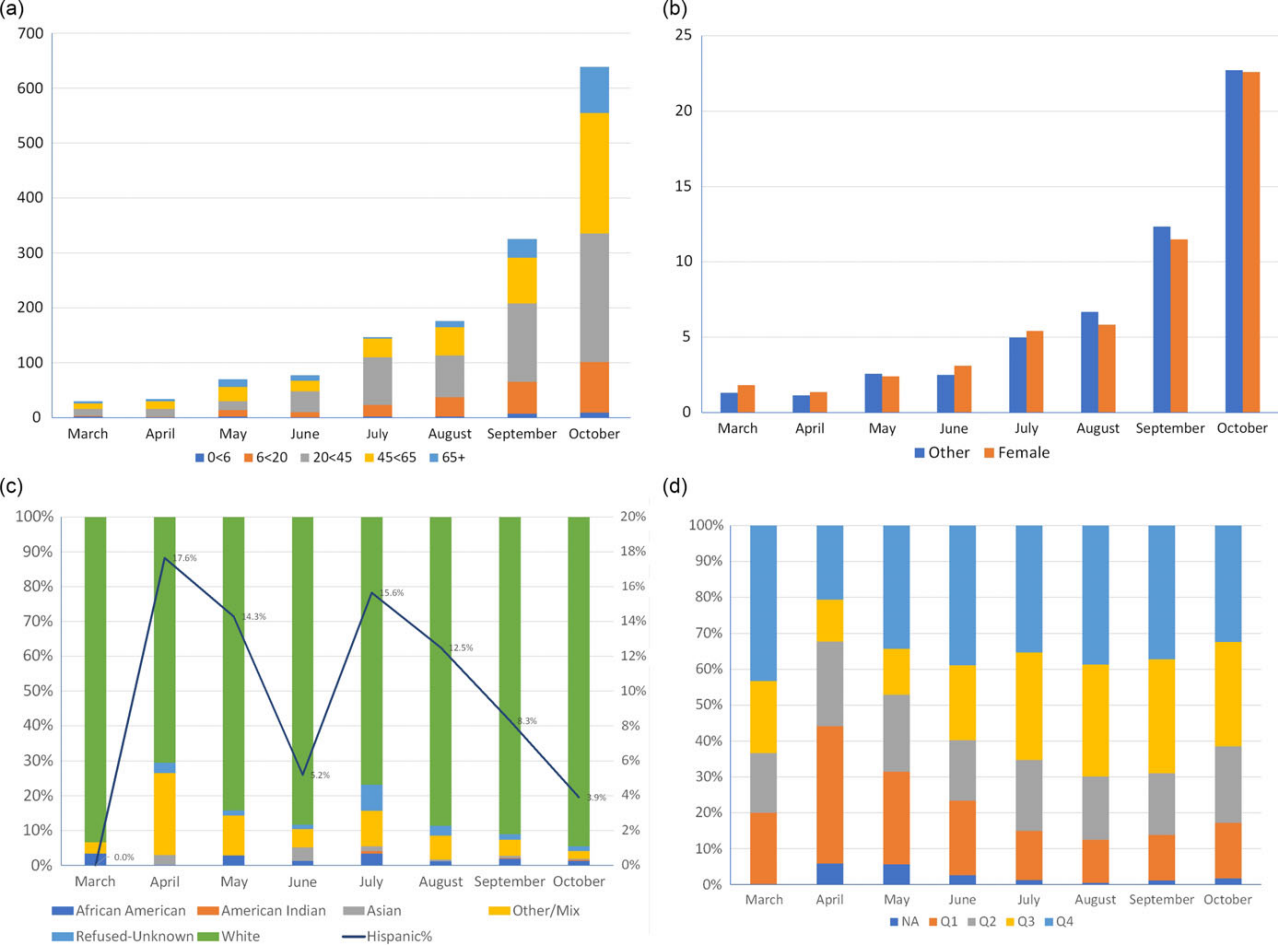
a) Count of Cases by Age Group by Month COVID-19 Four-County Rural Analysis Southeast Minnesota 3/11–10/31/2020. b) Cases per 100,000 Population per Day by Sex Rural Four-County Area Study. c) Share of Cases by Race and Ethnicity by Month COVID-19 Four-County Rural Analysis Southeast Minnesota 3/11–10/31/2020. d) Share of Cases by HOUSES Quartile COVID-19 Four-County Rural Analysis Southeast Minnesota 3/11–10/31/2020.

Figs. 2a–d show temporal (monthly) trends of COVID-19 cases in relation to demographics (2a for age, 2b for sex, 2c for race/ethnicity, and 2d for SES).

While there were some fluctuations of COVID-19 prevalence over time, the proportions by gender and age subgroups had similar temporal trends. The highest prevalence of COVID-19 was among 20–44 years of age (2.3%), followed by 45–65 (1.9%). The proportion of racial and ethnic minorities (12.2% of rural population, excluding unknown) among COVID-19 cases was 33.3% in April and 22.8% in July and dramatically decreased in August through October, to 7.0%. The proportion of Hispanic persons (4.7% of the rural population) among cases was as high as 17.6% of total cases in April and 14.8% of cases in July, falling to 3.9% of cases in October. Overall, Hispanic persons accounted for 7.8% of cases. White cases exceeded the White proportion of population (94.3%) only in October (94.6%). Despite disparities in COVID-19 cases, little difference in testing rates was found by race: 29.1% African American, 26.5% Hispanic, 26.1% Asian, 26.6% White, 27.5% American Indian, and 28.0% Other/Mixed race (24.7% unknown/refused). Positivity rates were higher among several minority groups than among NHWs, with rates of 5.9% for NHW versus 9.7% for African American, 8.8% for Other race/Mixed race, and 10.2% for Hispanic persons of any race.

The proportion of the HOUSES quartile Q1 (lowest SES) testing positive exceeded its share of population during the March to June period. The share of cases among persons in the lowest HOUSES quartile was lower for all remaining periods, ranging from 68% to 96% of the overall average. For the share of cases among the highest quartile (Q4) exceeded the average of all SES levels in all periods. Hotspots in the March through June period (see Supplement) included 3 large MHCs (2 in cities, 1 in a township) and 12 apartment complexes in cities, identified through aggregating cases by address and verifying the structure types at the addresses with more than 11 REP records.

Testing proportion varied with city population. In cities under 1000, 23.2% of the population was tested versus 30.2% for cities over 5000. Positivity rates in small cities ranged from 5.2% in cities with population over 5000 to 6.1% in cities under 1000. Township population size was related to testing but not positivity. The testing rate in townships over 1200 was 27.6% versus 21.8% in townships under 500. Cities had more cases per capita in March–June, then townships had more cases per capita until late October.

### Geospatial and Temporal Trends of COVID-19 in Rural Areas

**Fig. 3.**
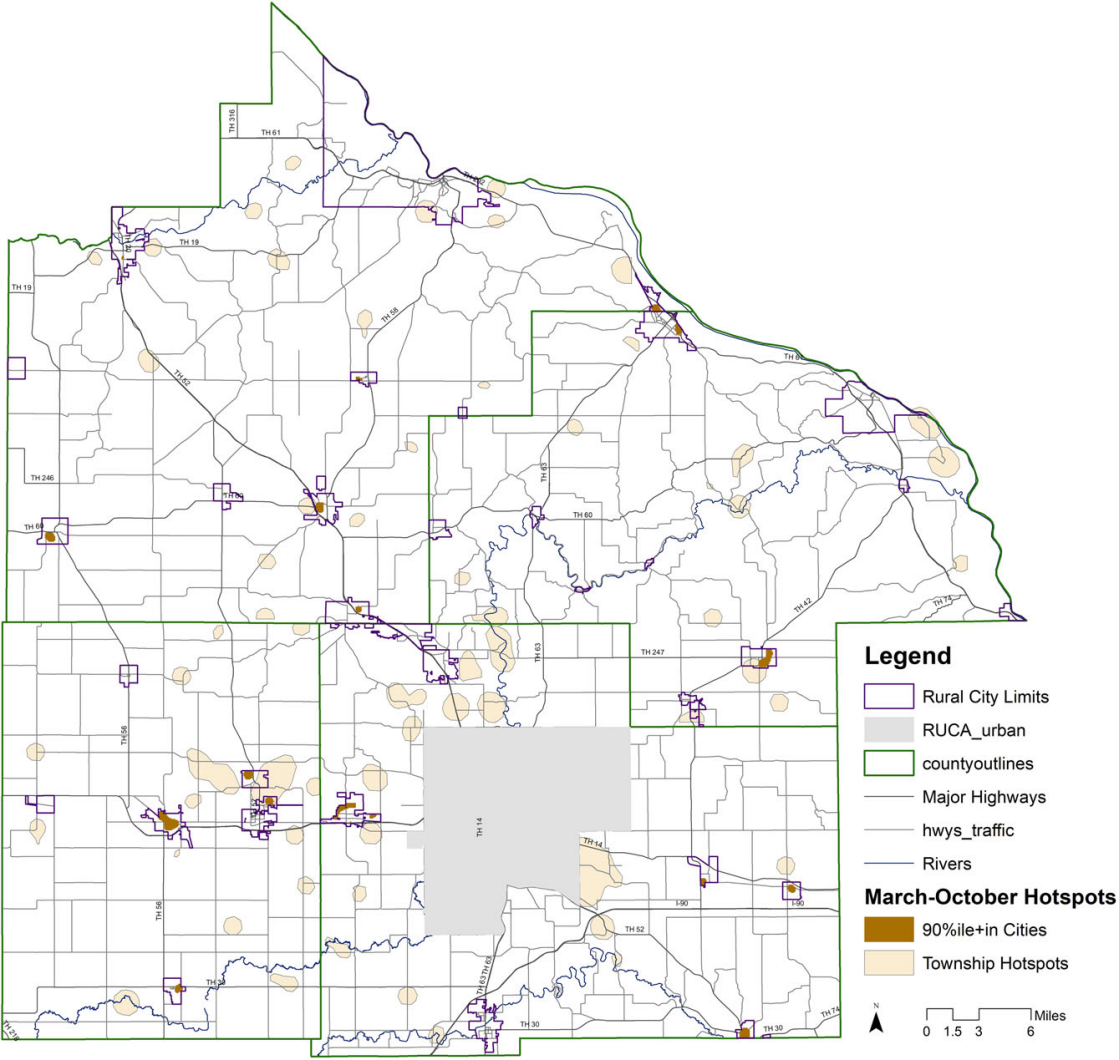
City and Township Cumulative Hotspots COVID-19 Four-County Rural Analysis Southeast Minnesota 3/11-10/31/2020.

Temporal geospatial analysis county maps are provided in the Supplement. Geospatial analysis results based on the entire study period are summarized in Fig. 3. Note that because hotspots are based on case density, overall March to October hotspots are somewhat influenced by high case numbers in September and October, when White, single-family, and higher SES households experienced high numbers of cases per capita. See maps in the Supplement for more temporal detail.

Hotspots occurring in cities tended to recur from month to month, while township hotspots were consistent only in concentrations of housing outside but adjacent to cities.

Temporal trends differed for all minorities compared to NHW persons, with cases per capita among the total racial minority population exceeding the rate for NHW persons in March through September (e.g., 16% higher in the July–August period), but lower by 1% in October. Hispanic persons of any race (the largest minority, 4,242 persons) per capita case rates were 2.2 times the NHW rate in March–June, 3.4 times in July–August, 1.9 times in September, and 0.8 times in October.

Finally, while all race, ethnic, and SES groups experienced an increase in cases in September and October, the large absolute increase in cases in October occurred chiefly due to an increase in cases among the White population, higher SES, and in single-family residential areas in rural communities.

### Comparison of Population Characteristics Inside and Outside Hotpots in Rural Areas

Rural city hotspots accounted for 33.3% (325/975) of cases and 14.1% of rural city population. As shown in Table 1, people living in city hotspots compared to other city residents tended to be younger, included a similar or higher proportion of minorities (except for African Americans), and higher SES. Hotspots and non-hotspots had the same proportion of HOUSES Q1 (lowest SES) residents through August at 23.2%, indicating the influence of a high proportion of higher SES cases in September and October.

**Table 1.** Characteristics of Residents of Cities in Rural Area

Variable	In hotspot	Outside hotspot	Odds ratio^*^ (95% CI)	P value^*^
Total N	8049	49,215		
Sex Female, N (%)	4242 (52.7%)	25,844 (52.5%)	0.992 (0.947–1.040)	0.75
Hispanic N (%)	546 (6.8%)	2423 (4.9%)	1.406 (1.277–1.548)	<0.0001
Race, N (%) African American	75 (0.9%)	697 (1.4%)	0.657 (0.517–0.834)	0.0006
Asian	67 (0.8%)	385 (0.8%)	1.061 (0.817–1.377)	0.66
Other/Mixed^**^	395 (4.9%)	2243 (4.6%)	1.309 (1.150–1.490)	<0.0001
Refusal/Unknown	105 (1.3%)	739 (1.5%)	1.079 (0.644–1.809)	0.77
White	7407 (92.0%)	45,151 (91.7%)	Reference	
Age, Median (IQR)	37.0 (17.9, 55.9)	40.5 (20.3, 61.1)	0.994 (0.994–0.995)	<0.0001
HOUSES, N (%) Q1	1363 (17.2%)	11,419 (23.8%)	0.559 (0.521–0.601)	<0.0001
Q2	1939 (24.4%)	13,149 (27.4%)	0.691 (0.647–0.738)	<0.0001
Q3	2339 (29.5%)	12,741 (26.5%)	0.860 (0.808–0.916)	<0.0001
Q4	2291 (28.9%)	10,737 (22.3%)	Reference	
Missing	117	1169		

* P values for testing association between variables and hotspot status (in hotspots), using logistic regression.

** Other/Mixed category includes other, mixed (2+ races), American Indian, and Hawaiian/Pacific Islander.

Rural township hotspots accounted for 48.8% of cases (277/568) and 27.1% of township population. As shown in Table 2, people living in township hotspots compared to other township residents tended to be younger, included a higher proportion of minorities, and were of higher SES.

**Table 2.** Characteristics of Residents of Townships in Rural Area

Variable	In hotspot	Outside hotspot	Odds ratio^*^ (95% CI)	P value^*^
Total N	6930	26,781		
Sex Female, N (%)	3504 (50.6%)	13,377 (49.9%)	0.976 (0.923–1.029)	0.36
Hispanic, N (%)	313 (4.5%)	990 (3.7%)	1.232 (1.082–1.403)	0.002
Race, N (%) African American	33 (0.5%)	84 (0.3%)	1.546 (1.033–2.315)	0.034
Asian	73 (1.1%)	133 (0.5%)	2.161 (1.622–2.878)	<0.0001
Other/Mixed^**^	252 (3.6%)	678 (2.5%)	1.542 (1.318–1.803)	<0.0001
Refusal/Unknown	68 (1.0%)	284 (1.1%)	0.859 (0.463–1.605)	0.63
White	6504 (93.9%)	25,602 (95.6%)	Reference	
Age, Median (IQR):	45.2 (21.3,61.1)	49.4 (23.8,64.6)	0.995 (0.994–0.996)	<0.0001
HOUSES, N (%) Q1	820 (11.9%)	2723 (10.1%)	1.003 (0.919–1.094)	0.95
Q2	863 (12.6%)	4894 (18.6%)	0.587 (0.541–0.638)	<0.0001
Q3	1888 (27.5%)	7706 (29.3%)	0.816 (0.766–0.870)	<0.0001
Q4	3303 (48.1%)	11,000 (41.8%)	Reference	
Missing	56	458		

* P values for testing association between variables and hotspot status (in hotspots), using logistic regression.

** Other/Mixed category includes other, mixed (2+ races), American Indian, and Hawaiian/Pacific Islander.

Table 3 compares city and township hotspots with city and township areas outside hotspots in terms of test positivity over time and the cases (positive tests) per 100,000 population per day for the five aggregated time periods in our analysis. In both cities and townships, and for all periods, hotspots had higher positivity levels and higher cases per 100,000 population. The rate of cases per capita increased over 15 times from March–June to late October.

**Table 3.** COVID19 Test Data for Cities and Townships in Rural Area*

Variable	City Hotspot	City Not Hotspot	Township Hotspot	Township Not Hotspot
Total N	8049	49,215	6930	26,781
March–June				
Tested (N)	731	5061	698	2255
Positive (N)	34	114	33	30
Positive % of Tested	4.65%	2.25%	4.73%	1.33%
Cases/100,000 population/day	4.02	2.21	4.54	1.07
July–August				
Tested (N)	590	3931	605	1754
Positive (N)	53	141	64	65
Positive % of Tested	8.98%	3.59%	10.58%	3.71%
Cases/100,000 population/day	10.62	4.62	14.90	3.91
September				
Tested (N)	396	2139	319	975
Positive (N)	70	124	61	70
Positive % of Tested	17.68%	5.80%	19.12%	7.18%
Cases/100,000 population/day	28.99	8.40	29.34	8.71
October 1–15				
Tested (N)	259	1298	193	624
Positive (N)	34	96	45	53
Positive % of Tested	13.13%	7.40%	23.32%	8.49%
Cases/100,000 population/day	28.16	13.00	43.29	13.19
October 16–31				
Tested (N)	260	1299	218	638
Positive (N)	85	179	74	73
Positive % of Tested	32.69%	13.78%	33.94%	11.44%
Cases/100,000 population/day	66.00	22.73	66.74	17.04

* Note: Positive tests (cases) are not weighted by level of testing.

## Discussion

The rural burden of COVID-19 is similar to that experienced by other populations experiencing health disparities. Our longitudinal geospatial analysis adds new information on geographic risk factors significantly related to the overall burden of COVID-19 and associated racial/ethnic and SES groups within rural communities depending on the timing of the pandemic. Geospatial analyses showed consistent hotspots in several cities and in a few areas of townships, even after adjusting for underlying population density. While other studies have reported county-level geographic clusters of COVID-19 cases [47,48], this study demonstrates the importance and utility of identifying geographic hotspots within counties. Hotspots significantly accounted for the COVID-19 burden in the rural areas of these four midwestern counties. To our knowledge, this is the first longitudinal geospatial analysis for COVID-19 epidemiology at a neighborhood level in rural counties in the Midwest region of the USA. As a practical matter of targeting preventive measures and interventions, the hotspots identified through this study establish a method to focus efforts in neighborhoods at higher risk. This may be especially important during periods in which pandemic surges make contact tracing difficult. Testing and tracing efforts could be guided by identifying hotspots within counties.

The areas identified as hotspots in our geospatial analysis reflected a broad range of neighborhoods in terms of SES depending on the timing of the pandemic, with lower SES and minority households especially affected early (March through June 2020) in the pandemic and higher SES households affected later (July through October). This observation is novel and has important implications for understanding and designing the public health interventions. In these four rural counties, the relationship between SES and race/ethnicity and risk of COVID-19 depends on geographic location and timing (whether in an early or later stage) of the pandemic. Results mirrored national trends in some respects and in the urban settings [49]. Significant disparities in the burden of COVID-19 occurred despite community factors mitigating health disparities in this region such as higher median family income than the national average. We found that for most months, racial and ethnic minority populations were disproportionately impacted by COVID-19, especially in the beginning of the pandemic and those residing in townships, even in rural areas where their share of population is low. These findings have implications for interventions regarding preventive measures and vaccination. During the early phases of a pandemic, community health interventions should focus on under-resourced populations. During later phases, broader segments of the population need to be engaged. Our study findings indicate that community health interventions and allocation of resources (e.g., public health education, testing/tracing, and vaccine roll out) could benefit from data on the geographic distribution and neighborhood characteristics of patients and populations in the context of timing of the pandemic, given the well-recognized health effects of the places in which people live [50] and other SDOH [47,48,51]. For example, the study demonstrates that given a sufficiently rapid turnaround from testing to geographic analysis, the findings could guide precise outreach and other interventions based on geographic hotspots, for example, to neighborhoods with housing types (e.g., apartment) associated with hotspots. In this sense, the study represents a proof of concept. Prompt development of analyses will enable an evolving response to evolving conditions. As an application in our study setting, a geospatial analysis-guided flu vaccine outreach team went out during COVID-19 pandemic to vaccinate target populations in hotspots with influenza vaccine (when COVID vaccine was not available) to avoid the burden of influenza for under-resourced populations who were already significantly affected by COVID-19. This strategy can be applied to COVID-19 vaccinations depending on the geographic vaccination rates (e.g., hotspots for low vaccine uptake).

Our study has important strengths. First, our study is a population-based study leveraging a geographically well-defined population, a self-contained health care environment, and the REP, an electronic data repository for our region. Second, our study is the first longitudinal temporal geospatial analysis for COVID-19 epidemiology at a neighborhood level in rural counties in the Midwest region. The prevalence of COVID-19 and the analysis of hotspots reflected the number of tests, population density, and household SES. Third, we believe the study methods are generalizable to other rural areas wherever address data can be found for tested persons and positive tests, regardless of the proportion of cases in the population, although with lower incidence, the bandwidth would need to be adjusted even in higher density areas. If address data are not available (e.g., where only the Zip code of the tests and cases is known), it would be possible to identify “hotspot” Zip codes, but with much less precision as to the neighborhood characteristics associated with the hotspots. In our mixed urban-rural area, at least, zip code areas might not be suitable as they are heterogeneous in terms of SES, housing type, race, ethnicity, density, rural-urban character, and other relevant factors.

Our study also has several limitations. First, residents of cities and townships of lower population were less likely to be tested. Although we adjusted our analysis to account for testing, there may have been unreported cases in these areas. Second, our reliance on the REP as the data set means that some COVID-19 tests and cases are likely missing from our analysis. In addition, geocoded REP records for those with research authorization covered 75.0% of rural residents in the four-county study area, ranging from 63.7% in Goodhue County to 93.2% in Olmsted County. Third, parts of our study setting have a high proportion of health care workers compared to other settings, which may affect the generalizability of our study. Fourth, our analysis was not tested for spatial autocorrelation. Fifth, non-residential disease transmission (e.g., at workplaces) was not accounted for. In addition, while we know from other sources that rural counties have a lower level of vaccination and from the masking study [12] that rural people who affiliate with the Republican Party have a higher tendency to resist masking, neither vaccination status, masking habits, or party affiliation are part of the data for this study. Finally, in low density areas in cities, the kernel density approach is influenced by positive cases outside the areas having high weighted case density.

In conclusion, COVID-19 cases among the rural populations increased significantly during the study period. In these four Minnesota counties, geographic factors (hotspots) significantly account for the overall burden of COVID-19 and associated racial/ethnic and SES disparities in rural areas depending on the timing of the pandemic. Results could more precisely guide community outreach efforts (e.g., target populations, public health education, testing/tracing, and vaccine roll out) to those residing in hotspots.

## Supplementary material

For supplementary material accompanying this paper visit https://doi.org/10.1017/cts.2021.885. click here to view supplementary material

## References

[1] Centers for Disease Control and Prevention. COVID data tracker. (https://covid.cdc.gov/covid-data-tracker/#datatracker-home)

[2] National Center for Advancing Translational Sciences. CTSA program rural health efforts. Last updated July 9, 2021. (https://ncats.nih.gov/ctsa/projects/RuralHealth)

[3] Temple KM . NIH National Center for Advancing Translational Sciences: involving rural America in research. The Rural Monitor, 2019. (https://www.ruralhealthinfo.org/rural-monitor/ncats-rural-research/)

[4] National Institute on Minority Health and Health Disparities. Overview. Last updated May 5, 2021. (https://www.nimhd.nih.gov/about/overview/)

[5] St Sauver JL , Grossardt BR , Yawn BP , et al. Data resource profile: the Rochester Epidemiology Project (REP) medical records-linkage system. International Journal of Epidemiology 2012; 41(6): 1614–1624. 23159830 10.1093/ije/dys195 PMC3535751

[6] Goetz S , Tian Z , Schmidt C , Meadowcroft D. Rural COVID-19 cases lag urban areas but are growing much more rapidly. NERCRD and Penn State University. NERCRD COVID-19 Issues Brief No. 2020-3, April 3, 2020. (https://aese.psu.edu/nercrd/publications/covid-19-issues-briefs/rural-covid-19-cases-lag-urban-areas-but-are-growing-much-more-rapidly)

[7] Souch JM , Cossman JS. A commentary on rural-Urban disparities in COVID-19 testing rates per 100,000 and risk factors. The Journal of Rural Health 2021; 37(1): 188–190. 32282964 10.1111/jrh.12450 PMC7262182

[8] Duca LM , Coyle J , McCabe C , McLean CA . COVID-19 Stats: COVID-19 Incidence,* by Urban-Rural Classification† - United States, January 22-October 31, 2020§. Morbidity and Mortality Weekly Report 2020; 69(46): 1753. 10.15585/mmwr.mm6946a6 PMC7676636 33211682

[9] Centers for Disease Control and Prevention. Covid data tracker: trends in COVID-19 cases and deaths in the United States, by county-level population factors. Published 2020. (https://covid.cdc.gov/covid-data-tracker//#pop-factors_7daynewcases)

[10] Khubchandani J , Sharma S , Price JH , Wiblishauser MJ , Sharma M , Webb FJ. COVID-19 vaccination hesitancy in the United States: a rapid national assessment. Journal of Community Health 2021; 46(2): 270–277. 33389421 10.1007/s10900-020-00958-x PMC7778842

[11] Kirzinger A , Muñana C , Brodie M . Vaccine hesitancy in rural America. KFF COVID-19 vaccine monitor web site. Kaiser Family Foundation, January 7, 2021. (https://www.kff.org/coronavirus-covid-19/poll-finding/vaccine-hesitancy-in-rural-america/)

[12] Maciejko L , Fox J , Steffens M , et al. Factors associated with willingness to wear a mask to prevent the spread of COVID-19 in a Midwestern community. Mayo Clinic 2021. 10.1016/j.pmedr.2021.101543 PMC8411589 34493965

[13] Minnesota Department of Health. Covid-19 Weekly Report. 2020.

[14] US Census Bureau. 2014-2018 American Community Survey 5-Year Estimates. 2019.

[15] Rocca WA , Grossardt BR , Brue SM , et al. Data resource profile: expansion of the rochester epidemiology project medical records-linkage system (E-REP). International Journal of Epidemiology 2018; 47(2): 368–368j. 29346555 10.1093/ije/dyx268 PMC5913632

[16] Kurani SS , McCoy RG , Lampman MA , et al. Association of neighborhood measures of social determinants of health with breast, cervical, and colorectal cancer screening rates in the US Midwest. JAMA Network Open 2020; 3(3): e200618–e200618. 32150271 10.1001/jamanetworkopen.2020.0618 PMC7063513

[17] US Department of Agriculture. US Department of griculture Rural-urban commuting area codes, 2019.

[18] US Census Bureau. 2015-2019 American community Survey 5-Year estimates. 2020.

[19] Wi C-I , Wheeler PH , Kaur H , Ryu E , Kim D , Juhn Y. Spatio-temporal comparison of pertussis outbreaks in Olmsted county, Minnesota, 2004-2005 and 2012: a population-based study. BMJ Open 2019; 9(5): e025521. 10.1136/bmjopen-2018-025521 PMC6530371 31110089

[20] Patel AA , Wheeler PH , Wi C-I , et al. Mobile home residence as a risk factor for adverse events among children in a mixed rural-urban community: a case for geospatial analysis. Journal of Clinical and Translational Science. 2020; 4(5): 443–450. 33244434 10.1017/cts.2020.34 PMC7681126

[21] Juhn YJ , Beebe TJ , Finnie DM , et al. Development and initial testing of a new socioeconomic status measure based on housing data. Journal of Urban Health : Bulletin of the New York Academy of Medicine. 2011; 88(5): 933–944. 21499815 10.1007/s11524-011-9572-7 PMC3191204

[22] Juhn YJ , Beebe TJ , Finnie DM , et al. Development and initial testing of a new socioeconomic status measure based on housing data. Journal of Urban Health 2011; 88(5): 933–944. 21499815 10.1007/s11524-011-9572-7 PMC3191204

[23] Rocca WA , Yawn BP , St Sauver JL , Grossardt BR , Melton LJ. History of the rochester epidemiology project: half a century of medical records linkage in a US population. Mayo Clinic Proceedings 2012; 87(12): 1202–1213. 23199802 10.1016/j.mayocp.2012.08.012 PMC3541925

[24] St. Sauver JL , Grossardt BR , Leibson CL , Yawn BP , Melton Iii LJ , Rocca WA. Generalizability of epidemiological findings and public health decisions: an illustration from the rochester epidemiology project. Mayo Clinic Proceedings. 2012; 87(2): 151–160. 22305027 10.1016/j.mayocp.2011.11.009 PMC3538404

[25] St. Sauver JL , Grossardt BR , Yawn BP , Melton LJ , Rocca WA. Use of a medical records linkage system to enumerate a dynamic population over time: the rochester epidemiology project. American Journal of Epidemiology. 2011; 173(9): 1059–1068. 21430193 10.1093/aje/kwq482 PMC3105274

[26] Bang DW , Manemann SM , Gerber Y , et al. A novel socioeconomic measure using individual housing data in cardiovascular outcome research. International Journal of Environmental Research and Public Health 2014; 11(11): 11597–11615. 25396769 10.3390/ijerph111111597 PMC4245632

[27] Ghawi H , Crowson CS , Rand-Weaver J , Krusemark E , Gabriel SE , Juhn YJ. A novel measure of socioeconomic status using individual housing data to assess the association of SES with rheumatoid arthritis and its mortality: a population-based case-control study. BMJ Open 2015; 5(4): e006469. 10.1136/bmjopen-2014-006469 PMC4420936 25926142

[28] Takahashi PY , Ryu E , Hathcock MA , et al. A novel housing-based socioeconomic measure predicts hospitalisation and multiple chronic conditions in a community population. Journal of Epidemiology and Community Health. 2016; 70(3): 286–291. 26458399 10.1136/jech-2015-205925 PMC4852846

[29] Ryu E , Juhn YJ , Wheeler PH , et al. Individual housing-based socioeconomic status predicts risk of accidental falls among adults. Annals of Epidemiology 2017; 27(7): 415–420.e412. 28648550 10.1016/j.annepidem.2017.05.019

[30] Wi CI , Gauger J , Bachman M , et al. Role of individual-housing-based socioeconomic status measure in relation to smoking status among late adolescents with asthma. Annals of Epidemiology 2016; 26(7): 455–460. 27266369 10.1016/j.annepidem.2016.05.001 PMC4958494

[31] Wi CI , St Sauver JL , Jacobson DJ , etal Ethnicity. Socioeconomic status, and health disparities in a mixed rural-Urban US community-Olmsted county, Minnesota. Mayo Clinic Proceedings 2016; 91(5): 612–622. 27068669 10.1016/j.mayocp.2016.02.011 PMC4871690

[32] Butterfield MC , Williams AR , Beebe T , et al. A two-county comparison of the HOUSES index on predicting self-rated health. Journal of Epidemiology and Community Health. 2011; 65(3): 254–259. 20439350 10.1136/jech.2008.084723 PMC3905443

[33] Harris MN , Lundien MC , Finnie DM , et al. Application of a novel socioeconomic measure using individual housing data in asthma research: an exploratory study. NPJ Primary Care Respiratory Medicine 2014; 24(1): 14018. 10.1038/npjpcrm.2014.18 PMC4498187 24965967

[34] Johnson MD , Urm SH , Jung JA , et al. Housing data-based socioeconomic index and risk of invasive pneumococcal disease: an exploratory study. Epidemiology and Infection. 2013; 141(4): 880–887. 22874665 10.1017/S0950268812001252 PMC3812670

[35] Hammer R , Capili C , Wi C-I , Ryu E , Rand-Weaver J , Juhn YJ. A new socioeconomic status measure for vaccine research in children using individual housing data: a population-based case-control study. BMC Public Health 2016; 16(1): 1–9. 27655468 10.1186/s12889-016-3673-x PMC5031352

[36] Stevens MA , Beebe TJ , Wi C II , Taler SJ , St. Sauver JL , Juhn YJ. HOUSES index as an innovative socioeconomic measure predicts graft failure among kidney transplant recipients. Transplantation 2020; 104(11): 2383–2392. 31985729 10.1097/TP.0000000000003131 PMC8159015

[37] Ryan CS , Juhn YJ , Kaur H , Wi CI , Ryu E , King KS , Lachance DH . Long-term incidence of glioma in Olmsted county, Minnesota, and disparities in postglioma survival rate: a population-based study. Neuro-Oncology Practice 2020; 7(3): 288–298. doi: 10.1093/nop/npz065 . 32537178 PMC7274190

[38] Thacher TD , Dudenkov DV , Mara KC , Maxson JA , Wi CI , Juhn YJ. The relationship of 25-hydroxyvitamin D concentrations and individual-level socioeconomic status. The Journal of Steroid Biochemistry and Molecular Biology 2020; 197(415–20): 105545. 31751783 10.1016/j.jsbmb.2019.105545 PMC7015787

[39] Christi A.Patten YJJ , Ryu Euijung , Wi Chung-Il , King Katherine S , Bublitz Josh T , Pignolo Robert J. Rural-Urban health disparities for mood disorders and obesity in a midwestern community. Journal of Clinical and Translational Science 2020;Accepted 10.1017/cts.2020.27 PMC7681122 33244429

[40] Barwise A , Wi CI , Frank R , et al. An innovative individual-Level socioeconomic measure predicts critical care outcomes in older adults: a population-Based study. Journal of Intensive Care Medicine 2020, 885066620931020. 10.1177/0885066620931020 PMC7759584 32583721

[41] Ryu E , Olson JE , Juhn YJ , et al. Association between an individual housing-based socioeconomic index and inconsistent self-reporting of health conditions: a prospective cohort study in the Mayo Clinic Biobank. BMJ Open 2018; 8(5): e020054. 10.1136/bmjopen-2017-020054 PMC5961601 29764878

[42] Barwise A , Juhn YJ , Wi CI , Novotny P , Jaramillo C , Gajic O , Wilson ME . An individual housing-based socioeconomic status measure predicts advance care planning and nursing home utilization. American Journal of Hospice and Palliative Medicine 2019; 36(5): 362–369. doi: 10.1177/1049909118812431. 30458635 PMC6946026

[43] Bjur KA , Wi CI , Ryu E , Crow SS , King KS , Juhn YJ. Epidemiology of children with multiple complex chronic conditions in a mixed Urban-Rural US community. Hospital Pediatrics. 2019; 9(4): 281–290. 30923070 10.1542/hpeds.2018-0091 PMC6434974

[44] Bjur KA , Wi CI , Ryu E , et al. Socioeconomic status, Race/Ethnicity, and health disparities in children and adolescents in a mixed rural-Urban community-Olmsted county, Minnesota. Mayo Clinic Proceedings 2019; 94(1): 44–53. 30611453 10.1016/j.mayocp.2018.06.030 PMC6360526

[45] Ryu E , Wi CI , Crow SS , et al. Assessing health disparities in children using a modified housing-related socioeconomic status measure: a cross-sectional study. BMJ Open 2016; 6(7): e011564. 10.1136/bmjopen-2016-011564 PMC4964248 27449892

[46] Lynch BA , Finney Rutten LJ , Jacobson RM , et al. Health care utilization by body mass index in a pediatric population. Academic Pediatrics. 2015; 15(6): 644–650. 26443036 10.1016/j.acap.2015.08.009 PMC4760684

[47] Emeruwa UN , Ona S , Shaman JL , et al. Associations between built environment, neighborhood socioeconomic status, and SARS-CoV-2 infection among pregnant women in New York City. JAMA. 2020; 324(4): 390–392. 32556085 10.1001/jama.2020.11370 PMC7303894

[48] Drew DA , Nguyen LH , Steves CJ , et al. Rapid implementation of mobile technology for real-time epidemiology of COVID-19. Science 2020; 368(6497): eabc0473–1367. 10.1126/science.abc0473 PMC7200009 32371477

[49] Juhn YJ , Wheeler P , Wi CI , et al. Role of geographic risk factors in COVID-19 epidemiology: longitudinal geospatial analysis, Mayo Clinic Proceedings: Innovations, Quality & Outcomes, 2021. 10.1016/j.mayocpiqo.2021.06.011 PMC8272975 34308261

[50] Baum A , Wisnivesky J , Basu S , Siu AL , Schwartz MD. Association of geographic differences in prevalence of uncontrolled chronic conditions with changes in individuals’ likelihood of uncontrolled chronic conditions. JAMA. 2020; 324(14): 1429–1438. 33048153 10.1001/jama.2020.14381 PMC8094427

[51] Rasmussen SA , Khoury MJ , del Rio C. Precision public health as a key tool in the COVID-19 response. JAMA 2020; 324(10): 933–934. doi: 10.1001/jama.2020.14992 . 32805001

