# Supplementary material for: Role of geographic risk factors and social determinants of health in COVID-19 epidemiology: Longitudinal geospatial analysis in a midwest rural region
Source: J Clin Transl Sci. 2021 Dec 27;6(1):e51. doi: 10.1017/cts.2021.885 (PMC9108006; doi:10.1017/cts.2021.885)

SUPPLEMENT – Geospatial analysis of COVID-19 in a Midwest rural region

1. Supplementary Table 1 shows the cases per 100,000 population per day for each period of the pandemic, by HOUSES quartile, excluding population with missing HOUSES values. The table shows the shift in impact on population by SES from the early phase to later phases.

| **Supplementary Table 1: Number of cases per 100,000 population per day by Period and Jurisdiction Type** | | | | | |
| --- | --- | --- | --- | --- | --- |
|  | Mar-Jun | Jul-Aug | Sep | E-Oct | L-Oct |
| **Cities** | | | | | |
| Q1 N=12,782 | 3.20 | 4.04 | 8.08 | 11.47 | 27.87 |
| Q2 N=15,088 | 1.96 | 4.49 | 9.06 | 12.81 | 27.34 |
| Q3 N=15,080 | 1.77 | 7.38 | 12.38 | 20.34 | 28.60 |
| Q4 N=13,028 | 2.85 | 6.07 | 16.37 | 15.35 | 32.62 |
| **Townships** | | | | | |
| Q1 N=3,543 | 2.69 | 4.10 | 9.41 | 11.29 | 24.70 |
| Q2 N=5,757 | 1.65 | 5.04 | 8.69 | 22.00 | 23.88 |
| Q3 N=9,594 | 0.69 | 5.04 | 16.33 | 16.68 | 30.62 |
| Q4 N=14,303 | 2.33 | 8.01 | 13.28 | 22.84 | 26.22 |
| **All Rural** | | | | | |
| Q1 N=16,325 | 3.09 | 4.05 | 8.37 | 11.43 | 27.18 |
| Q2 N=20,845 | 1.87 | 4.64 | 8.95 | 15.35 | 26.39 |
| Q3 N=24,674 | 1.35 | 6.47 | 13.91 | 18.91 | 29.38 |
| Q4 N=27,331 | 2.58 | 7.08 | 14.76 | 19.27 | 29.27 |

1. The operational definition of “rural” includes every area outside RUCA Class 1.1.


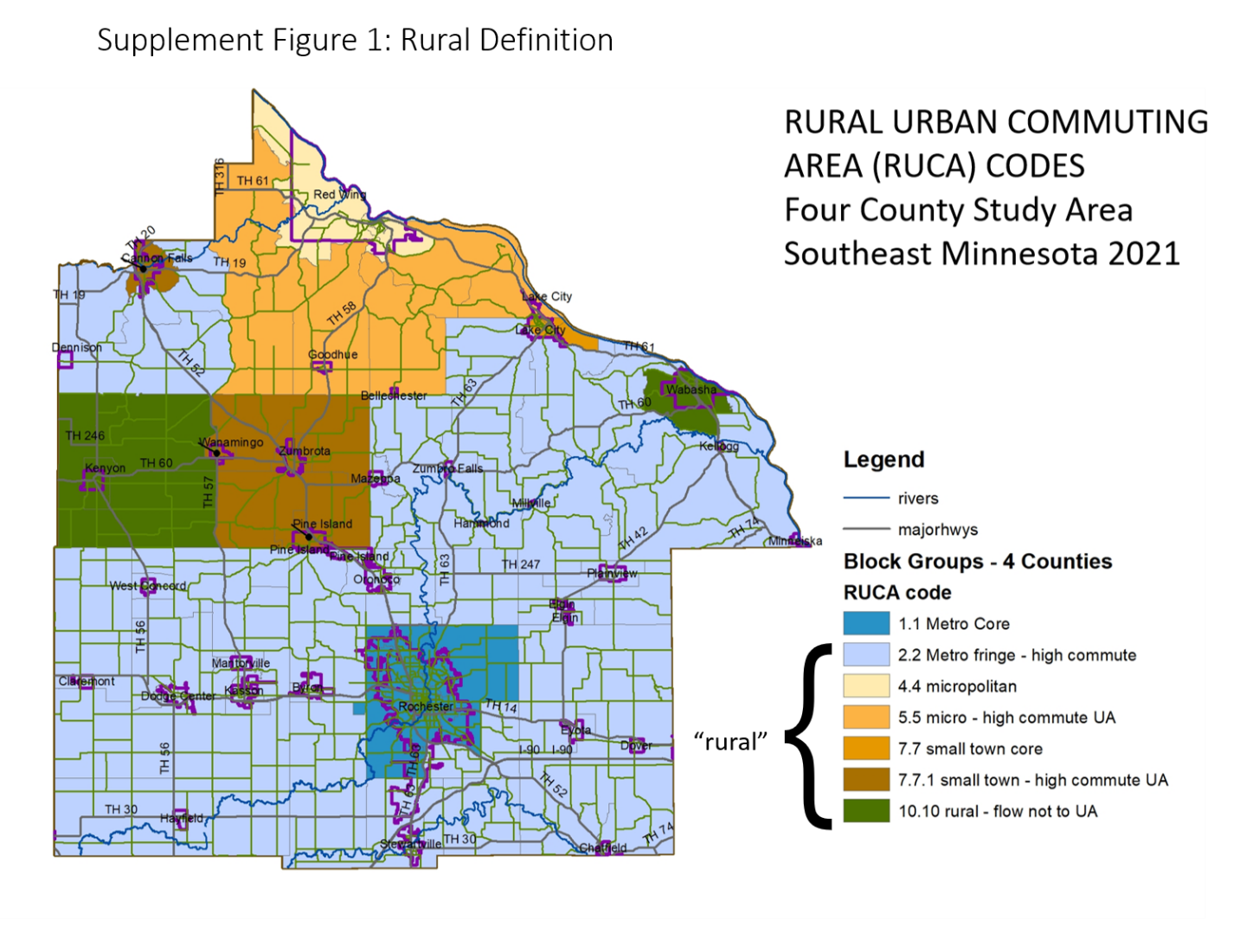


1. Figures 2a-2d, 3a-3d, 4a-4d, 5a-5d, and 6a-6d show the progression of hotspots in the rural areas of each of the four counties in the study. There were very few township hotspots in the earlier periods, with more extensive hotspots in later months for all counties. While city hotspots tended to occur in consistent areas for many cities, only a few of the township hotspots occurred in consistent locations over the periods of the study.


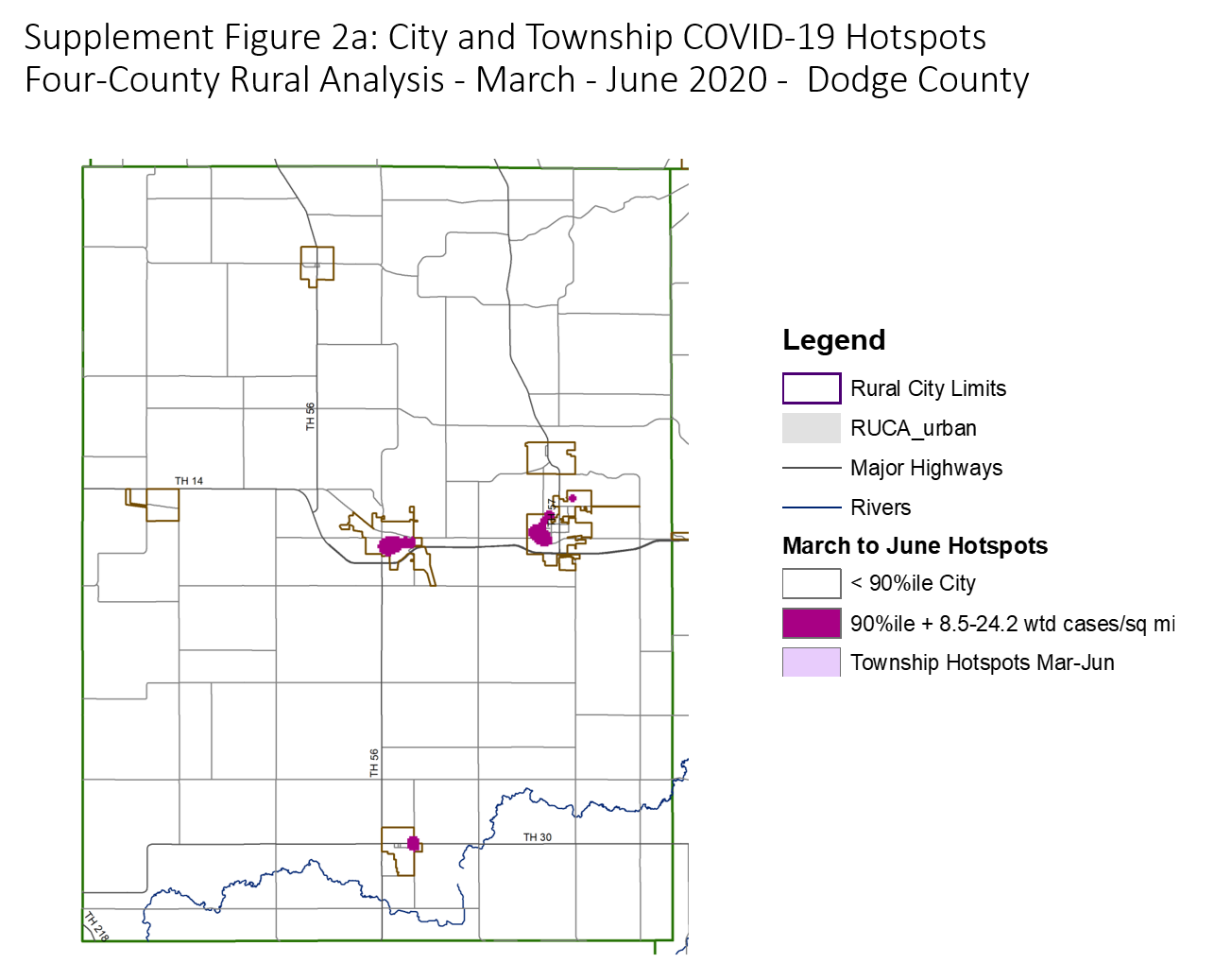

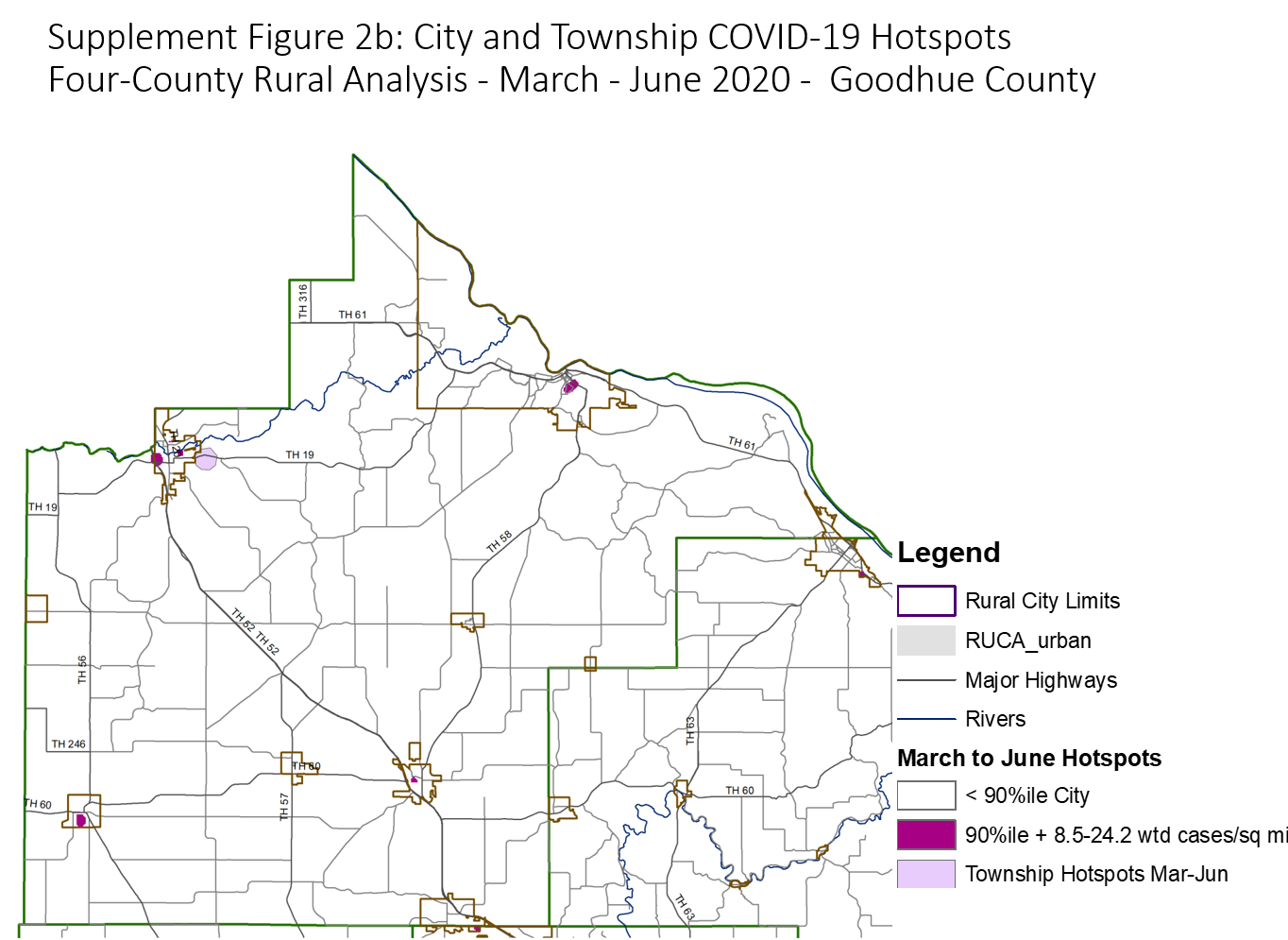

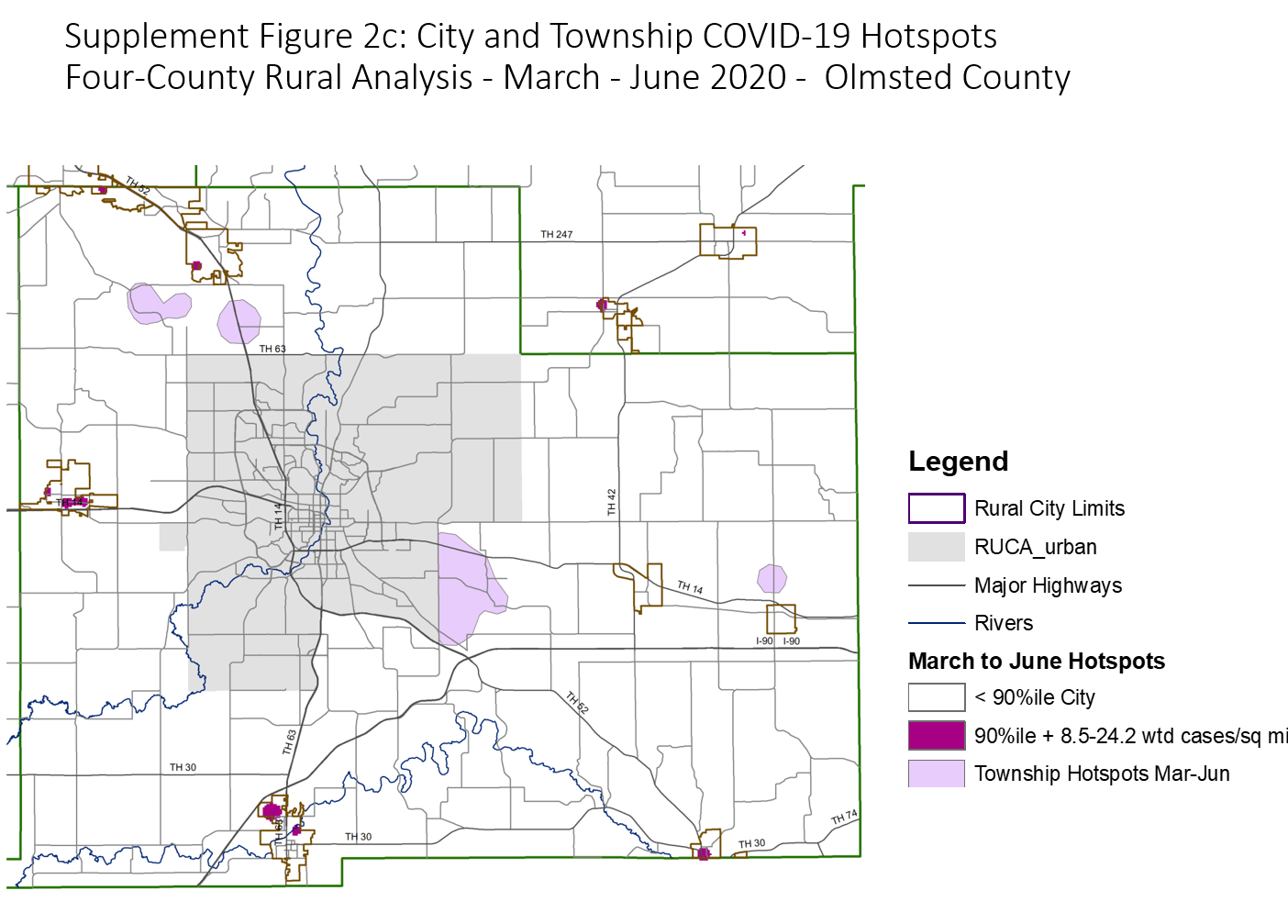

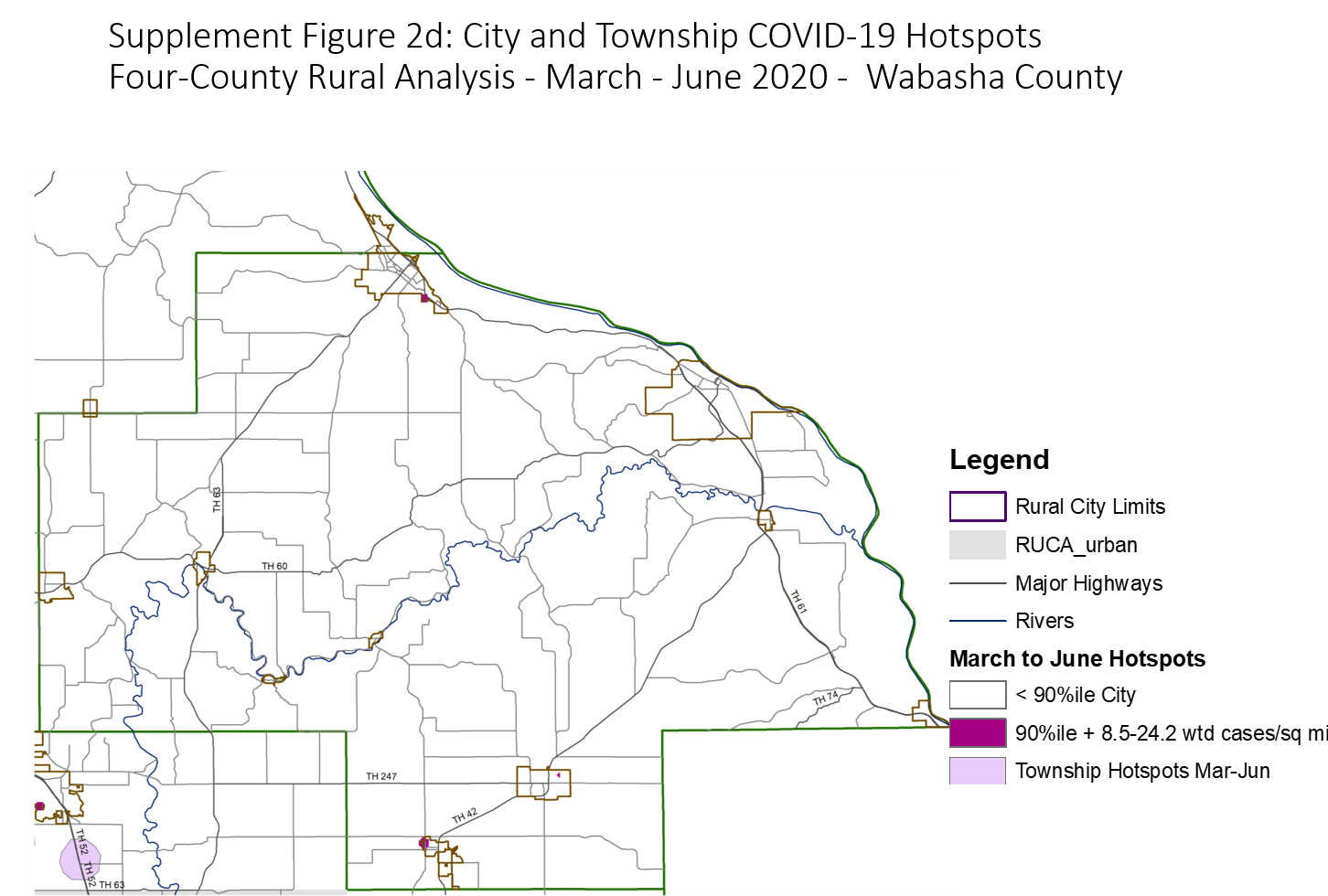

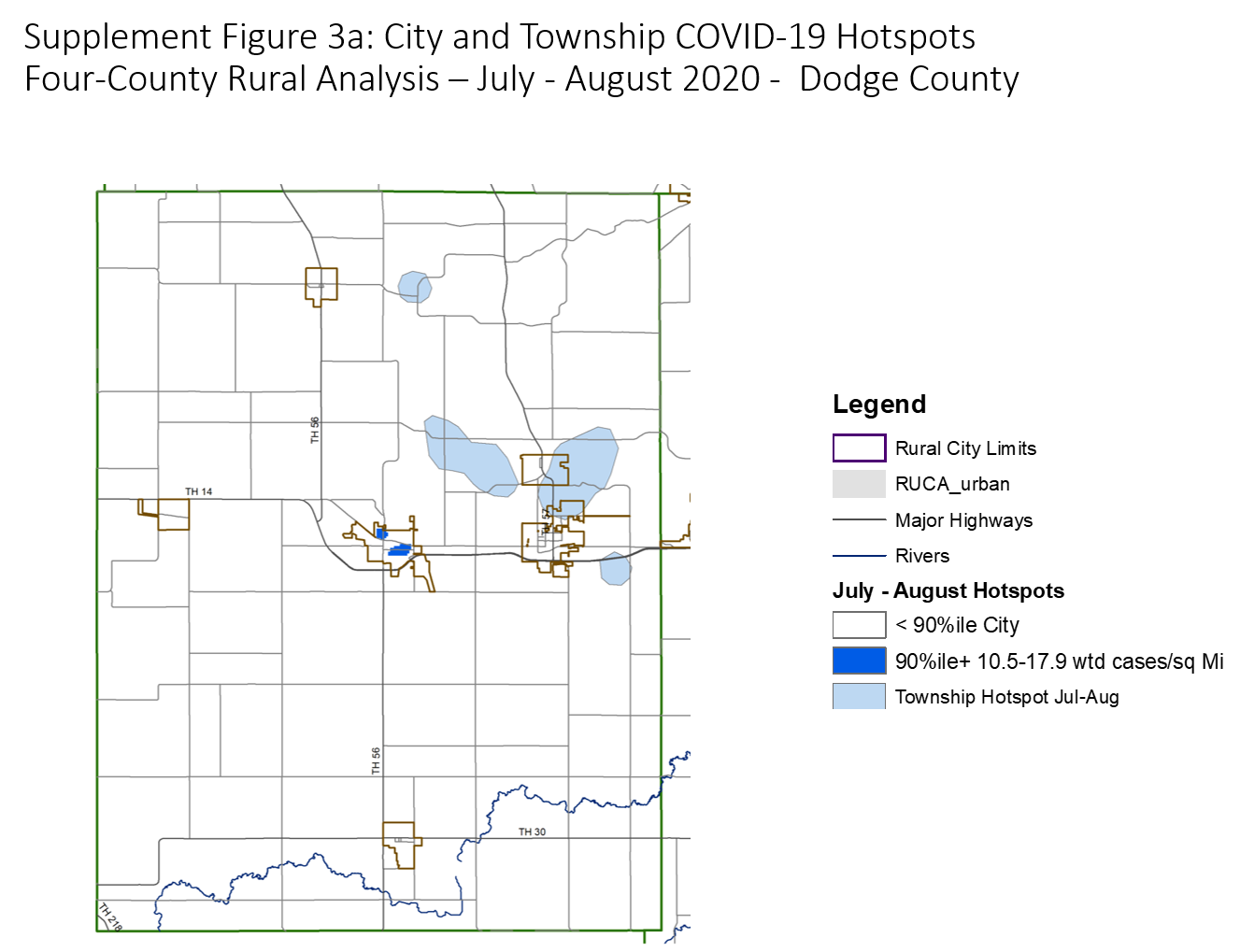

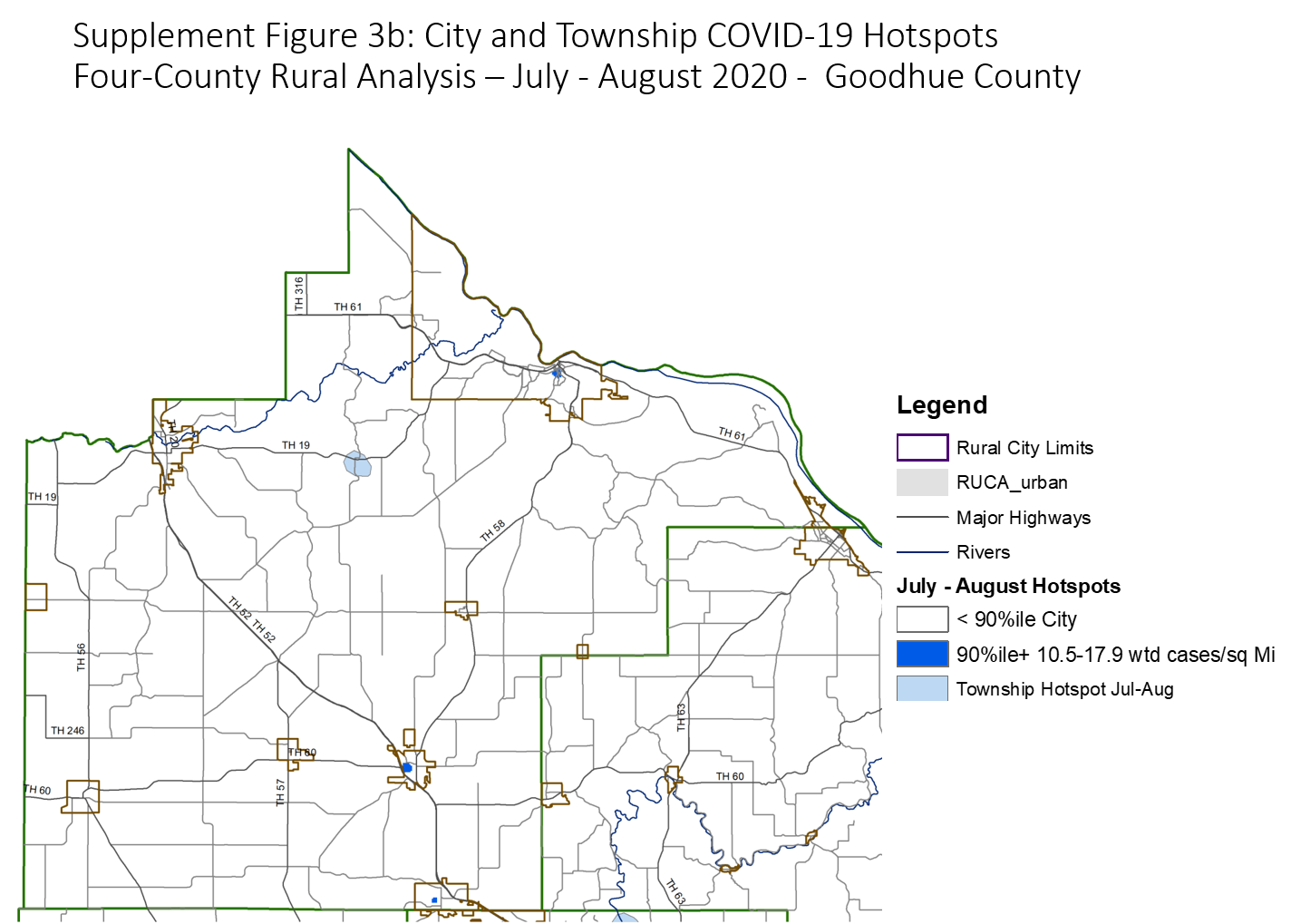

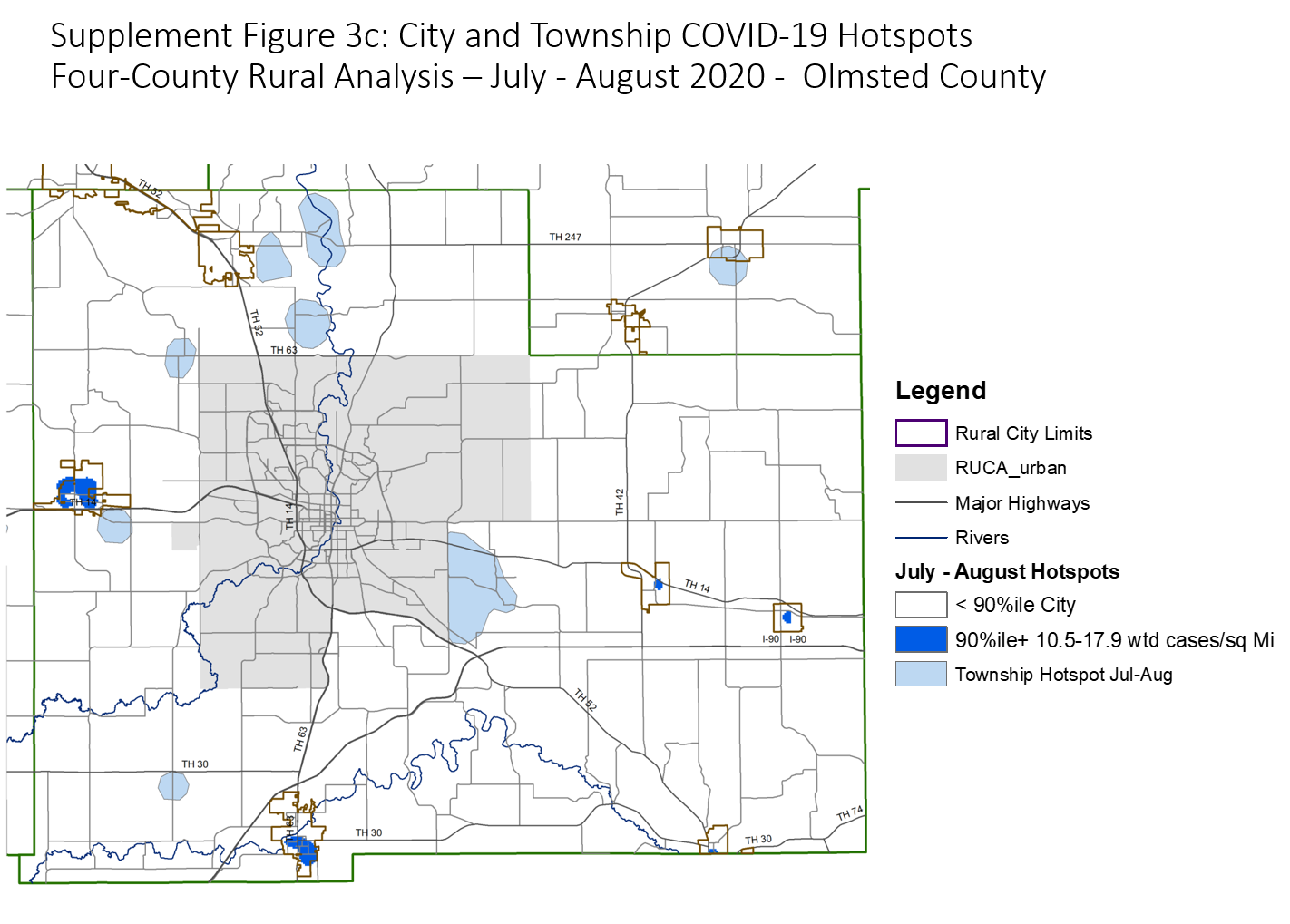

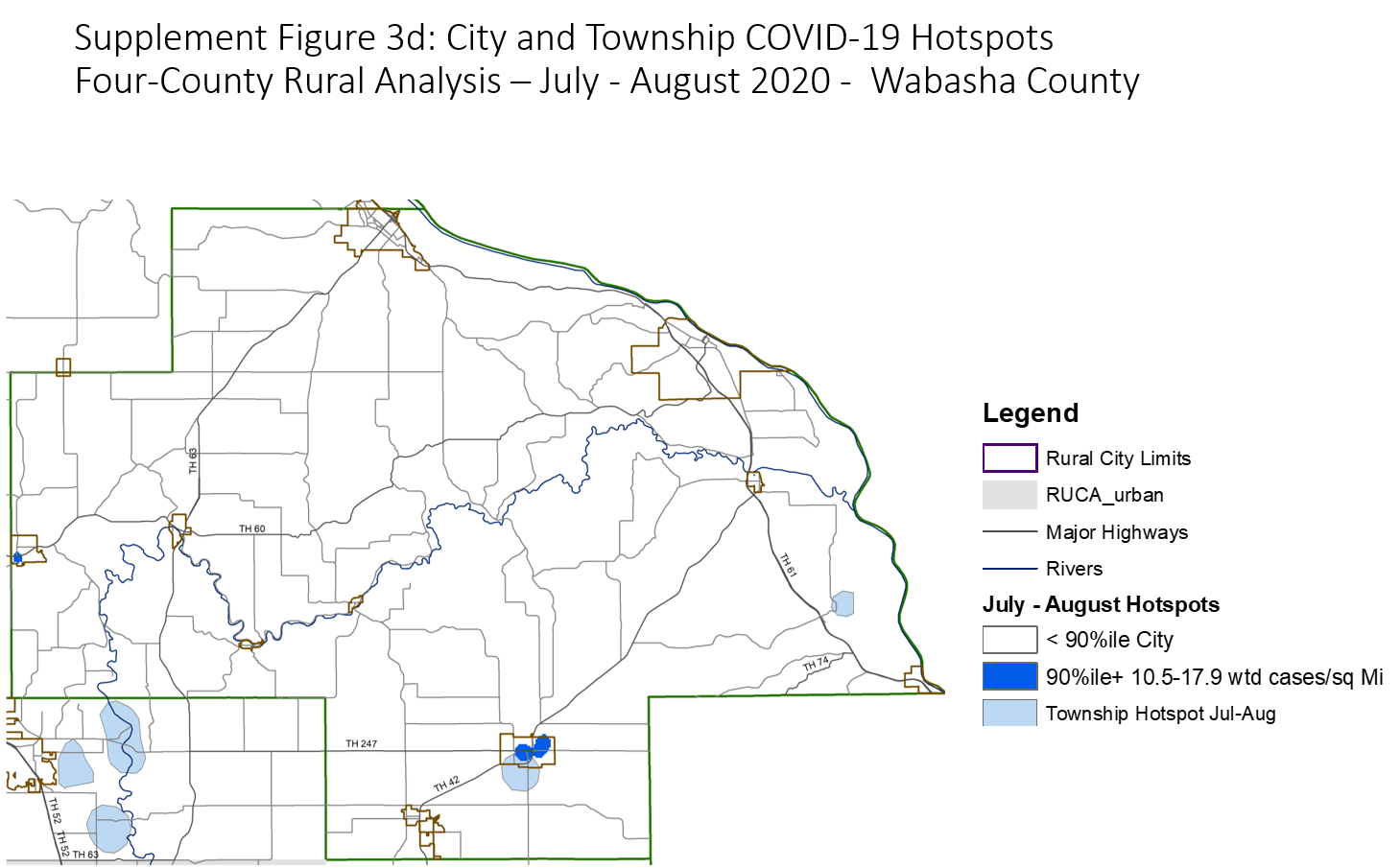

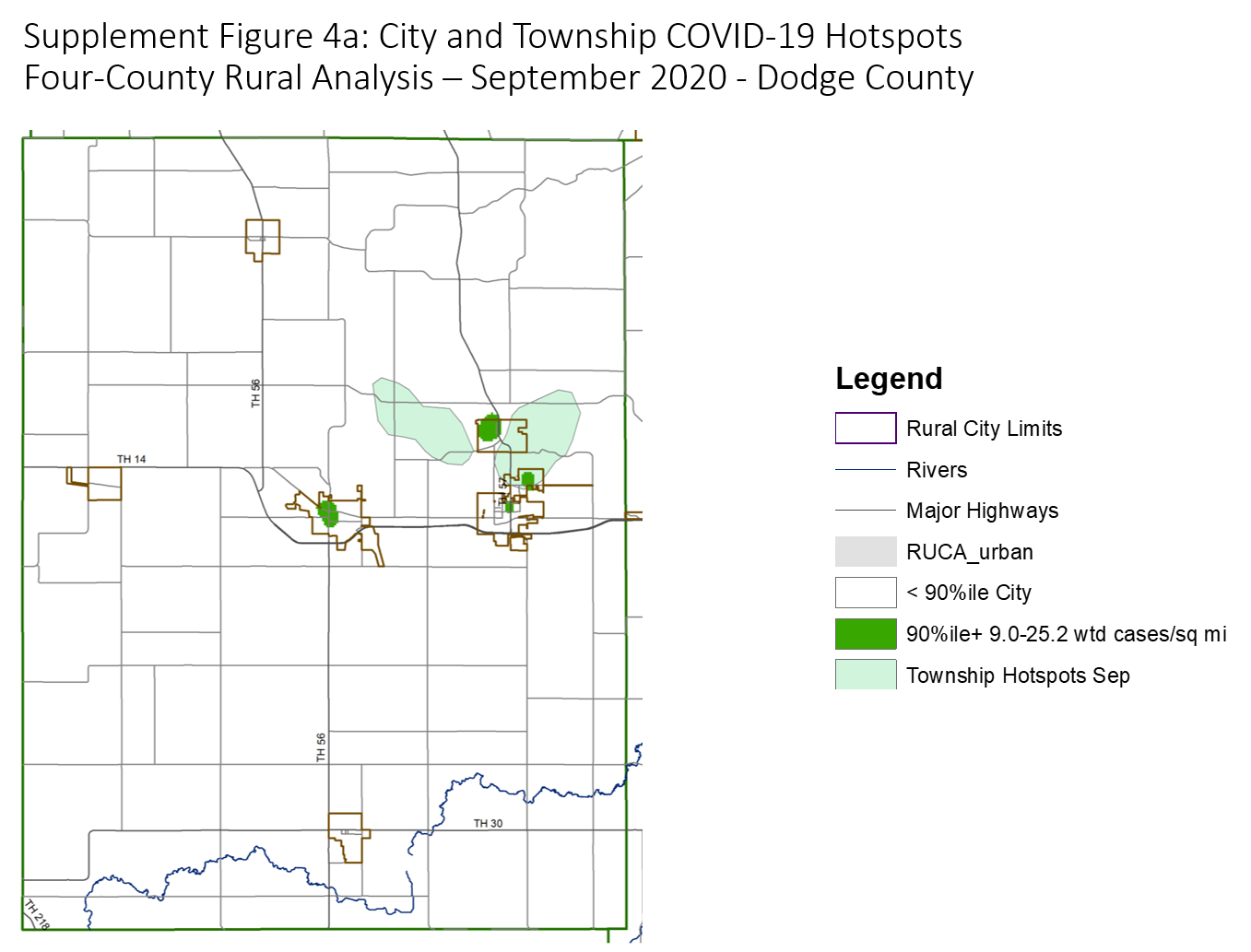

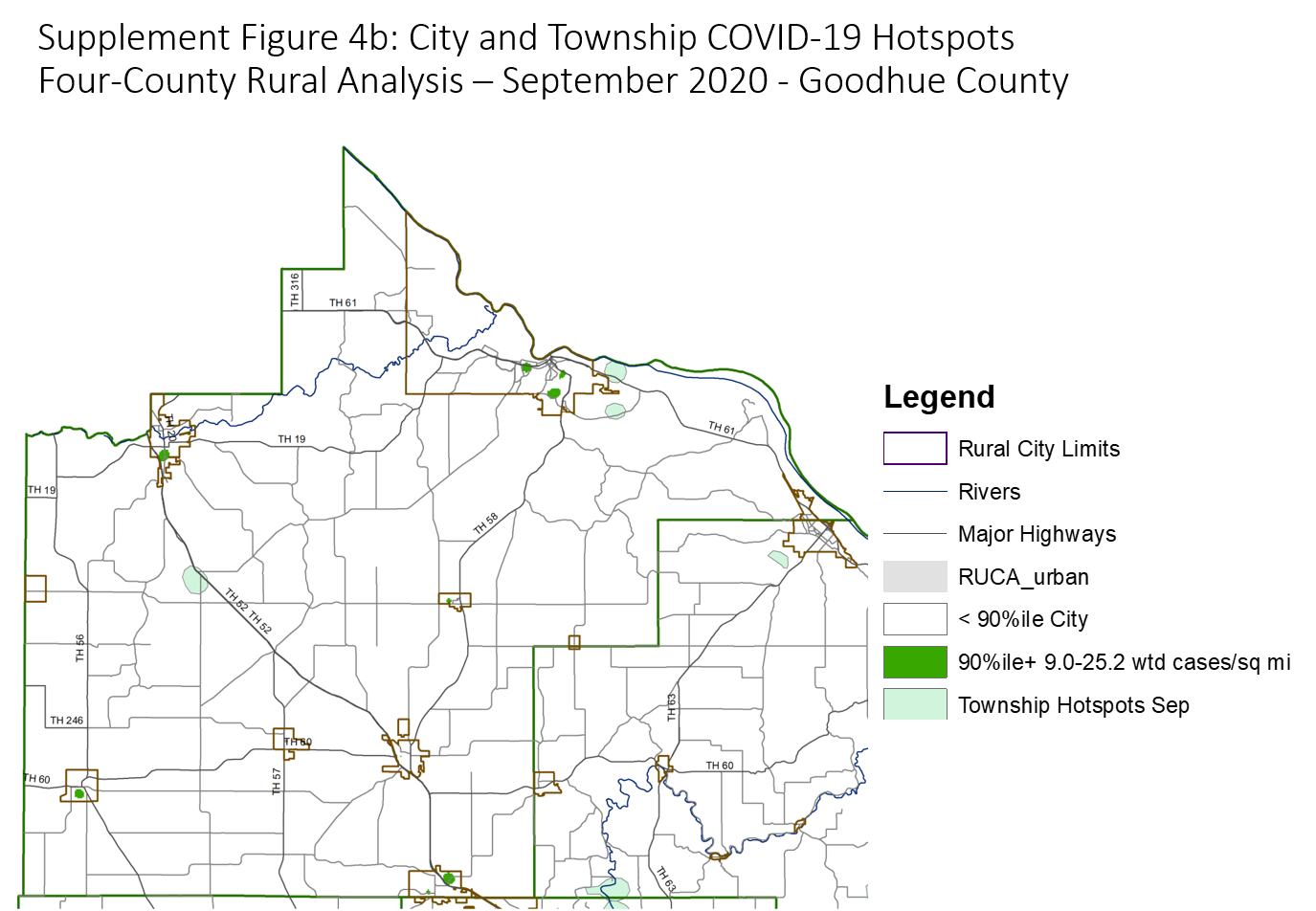

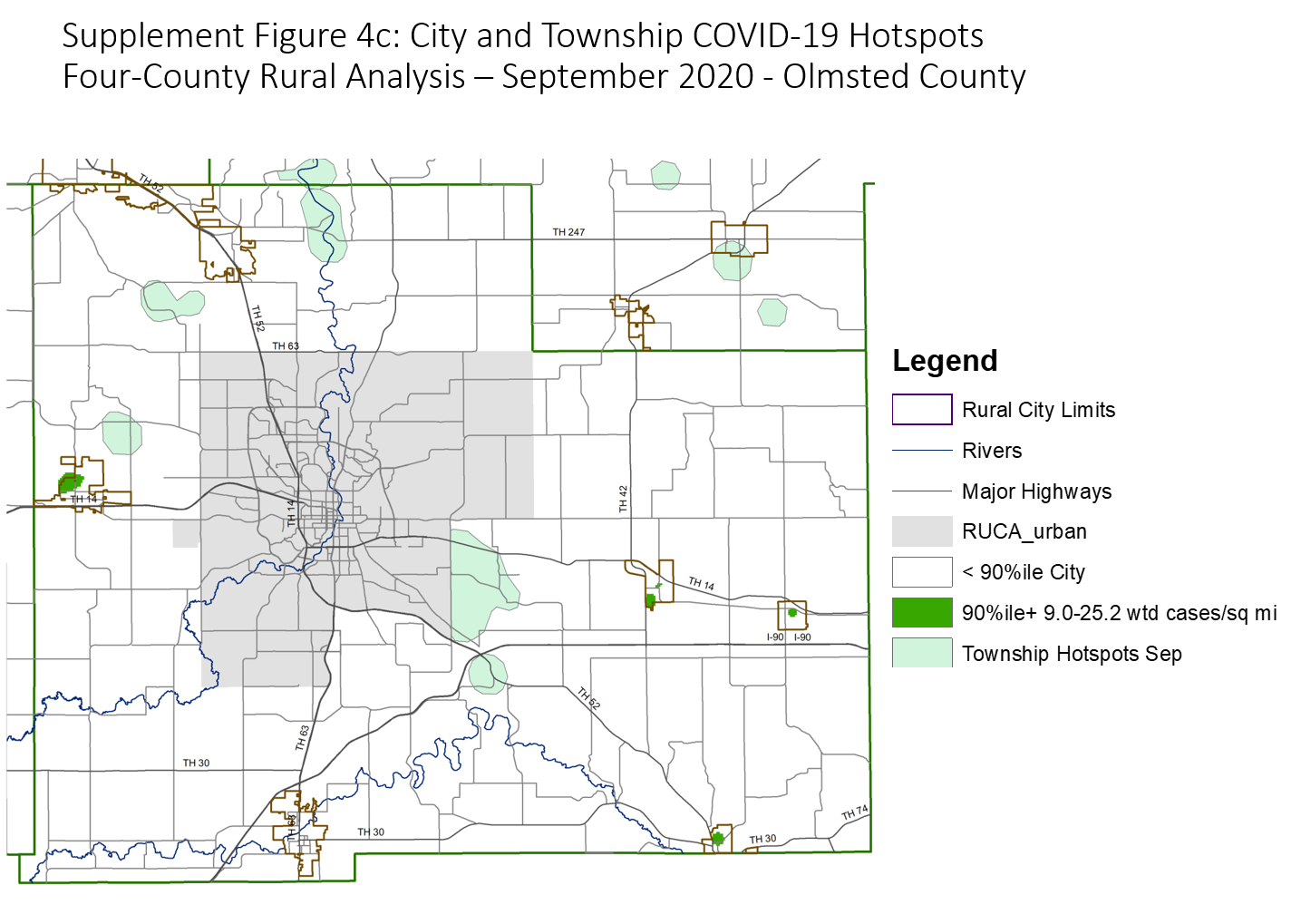

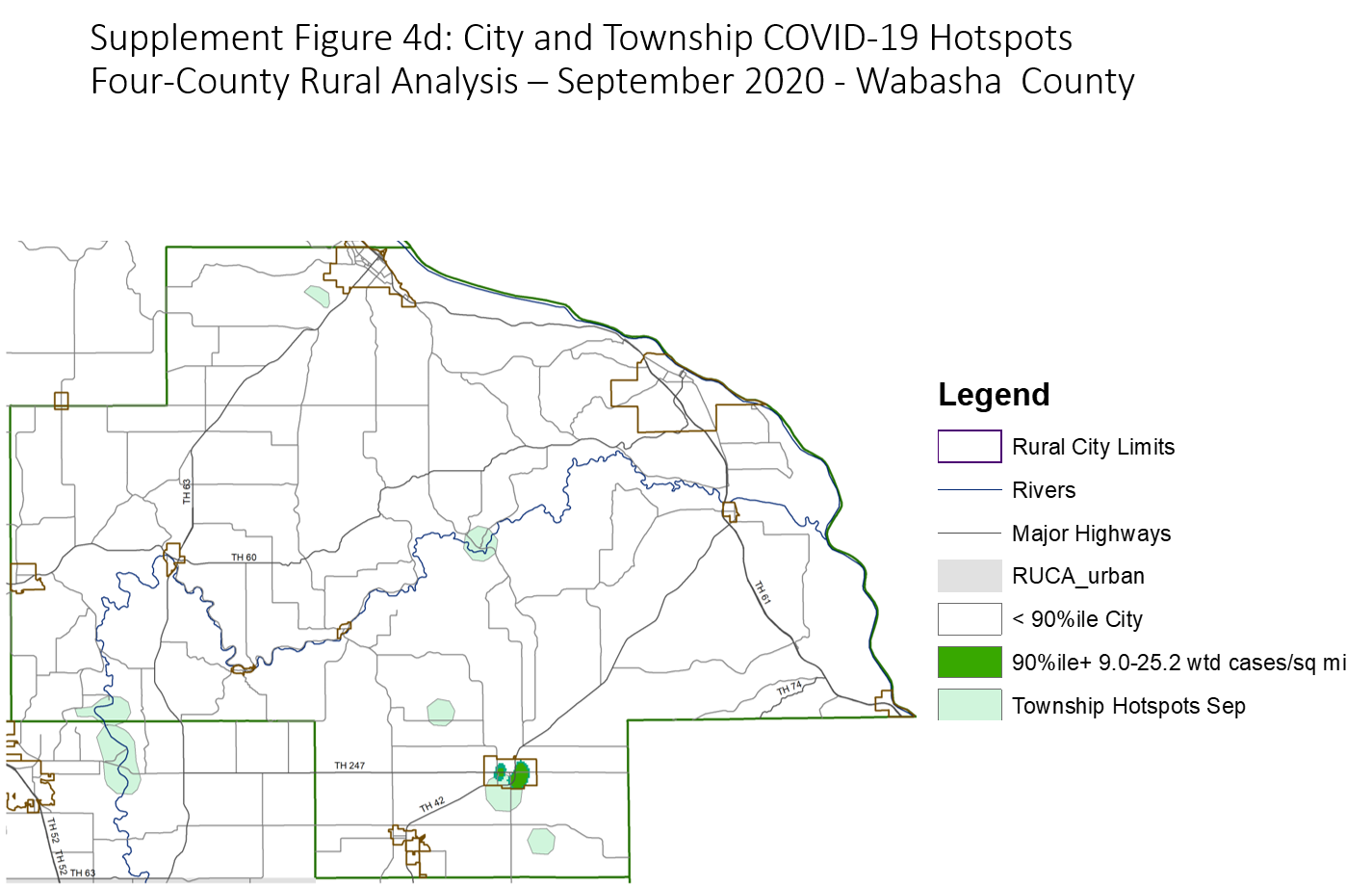

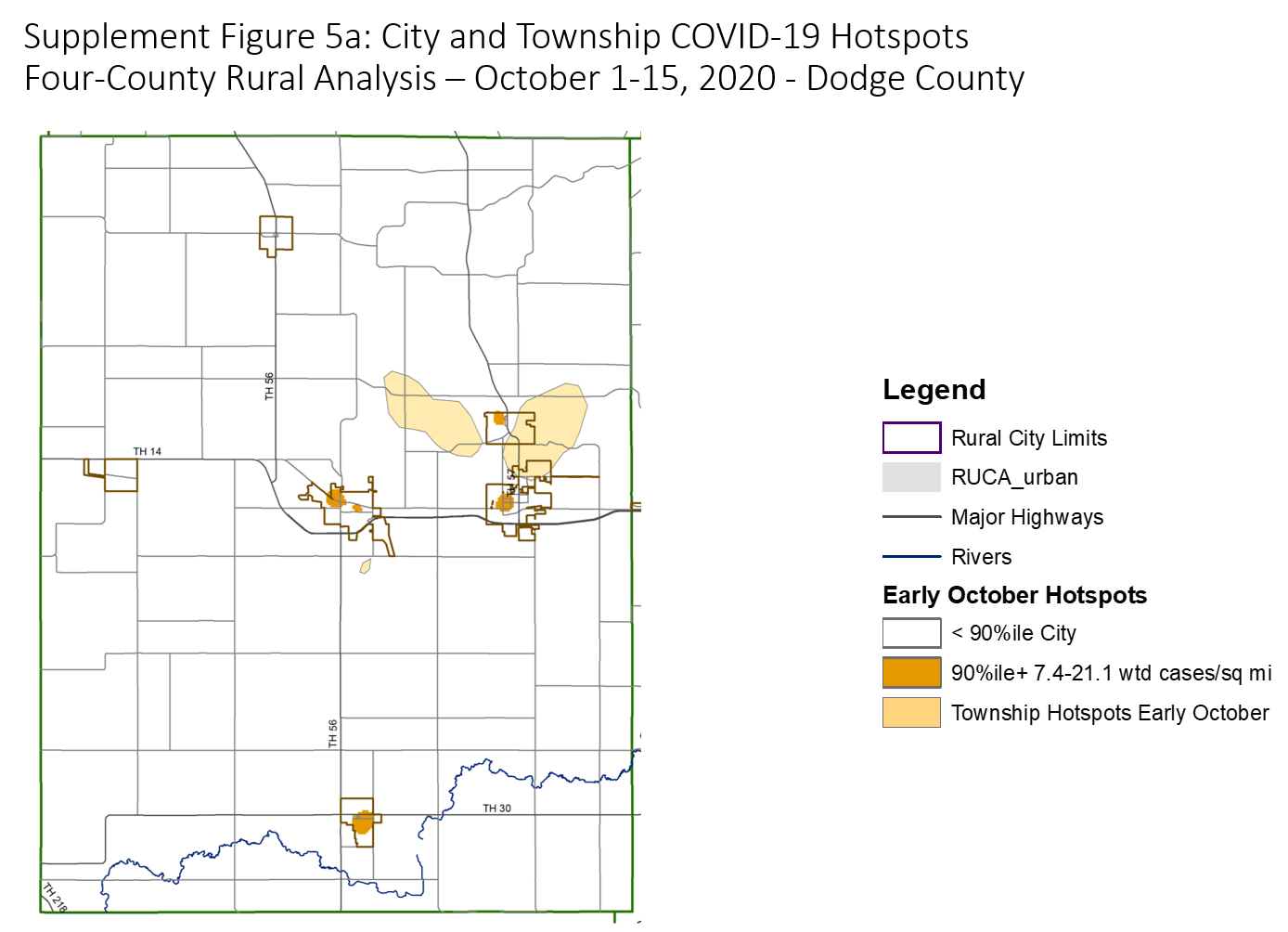

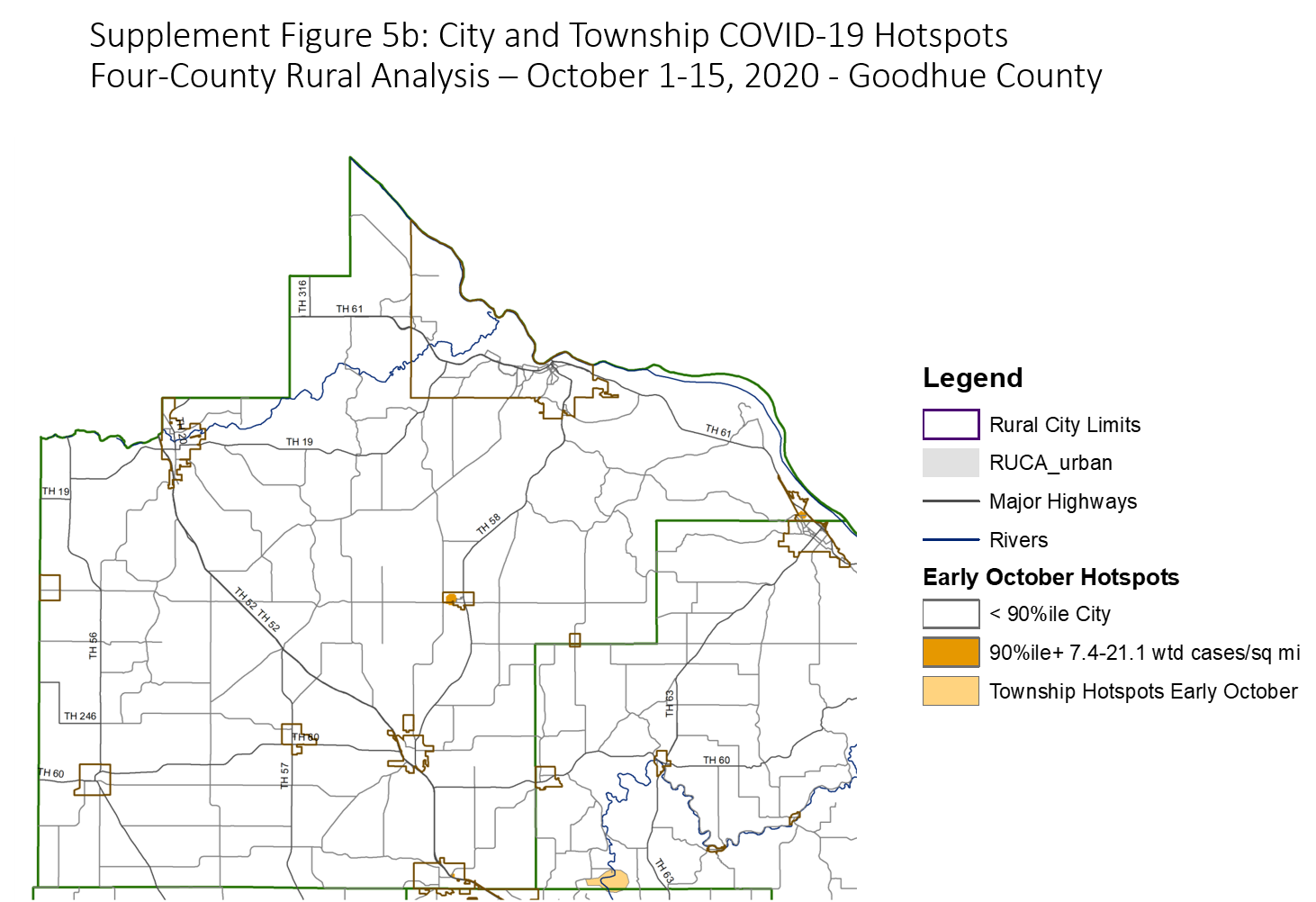

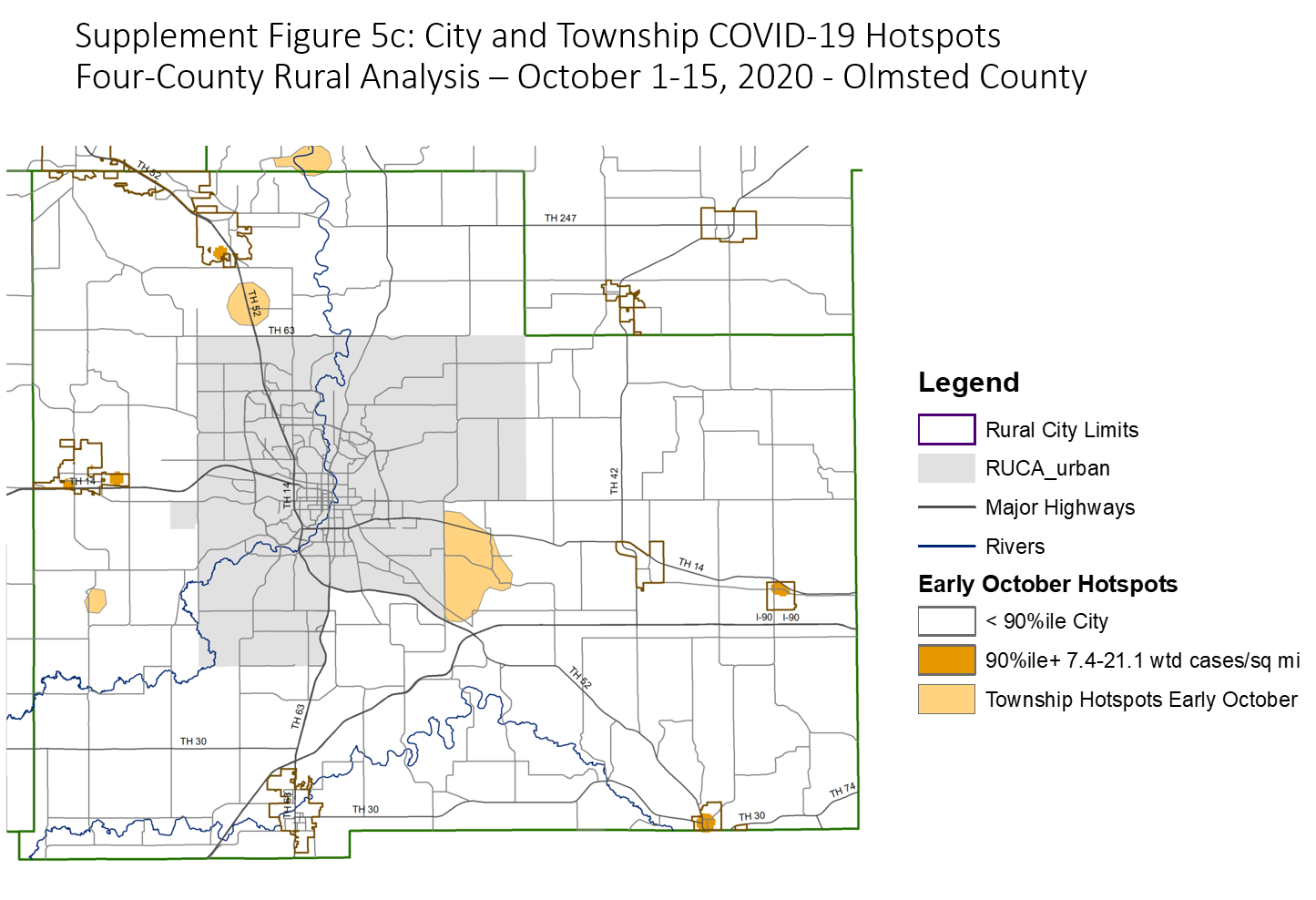

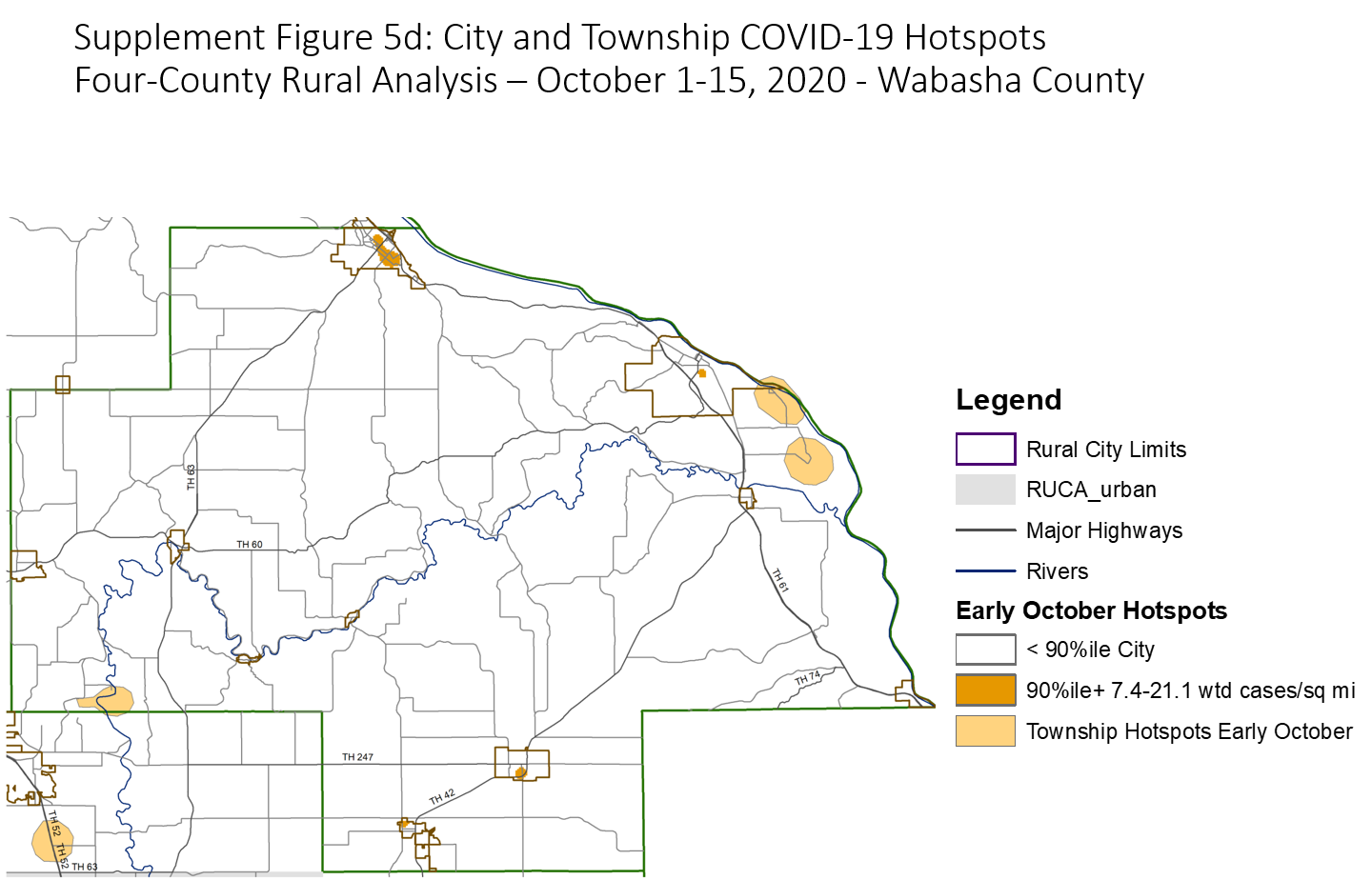

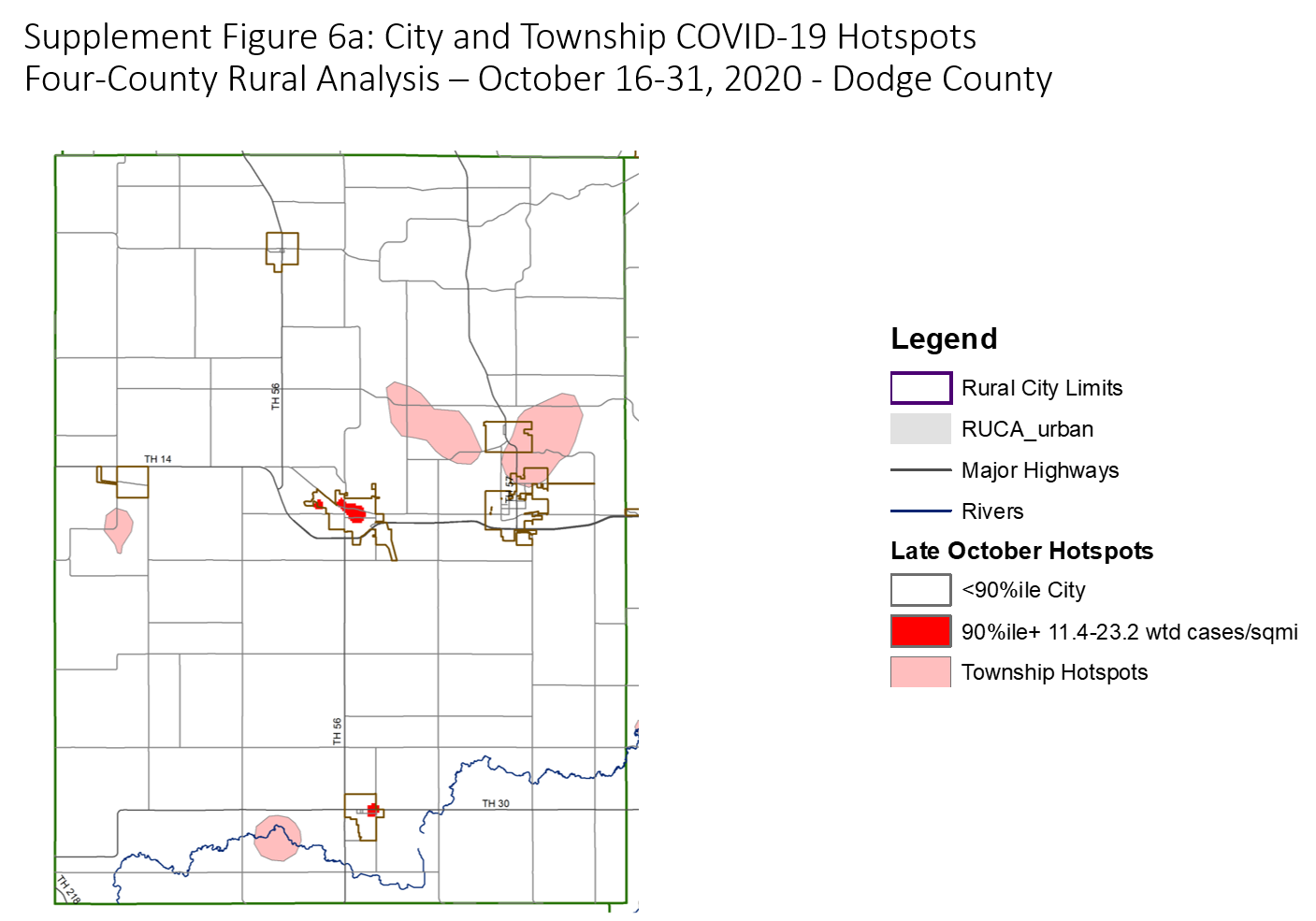

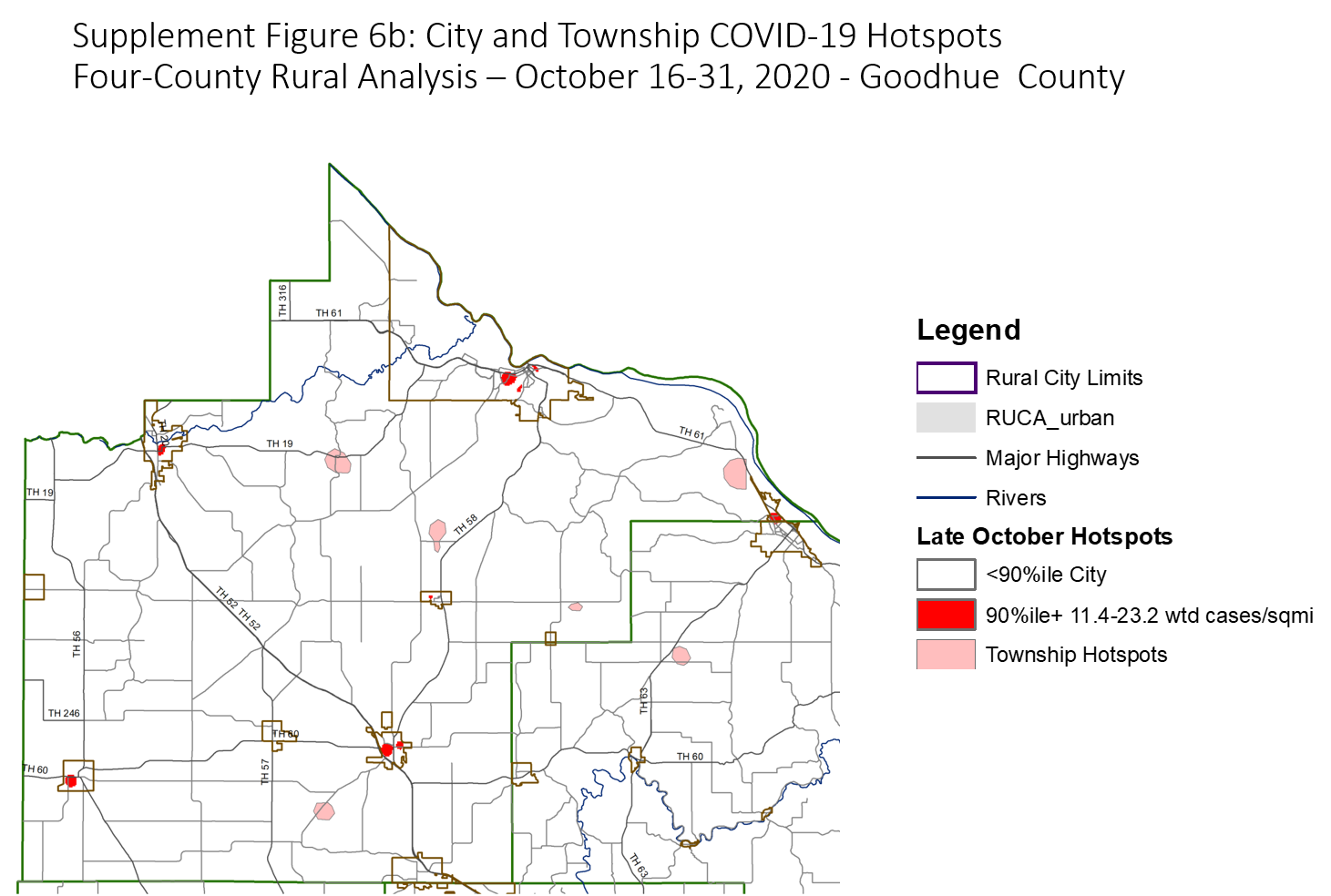

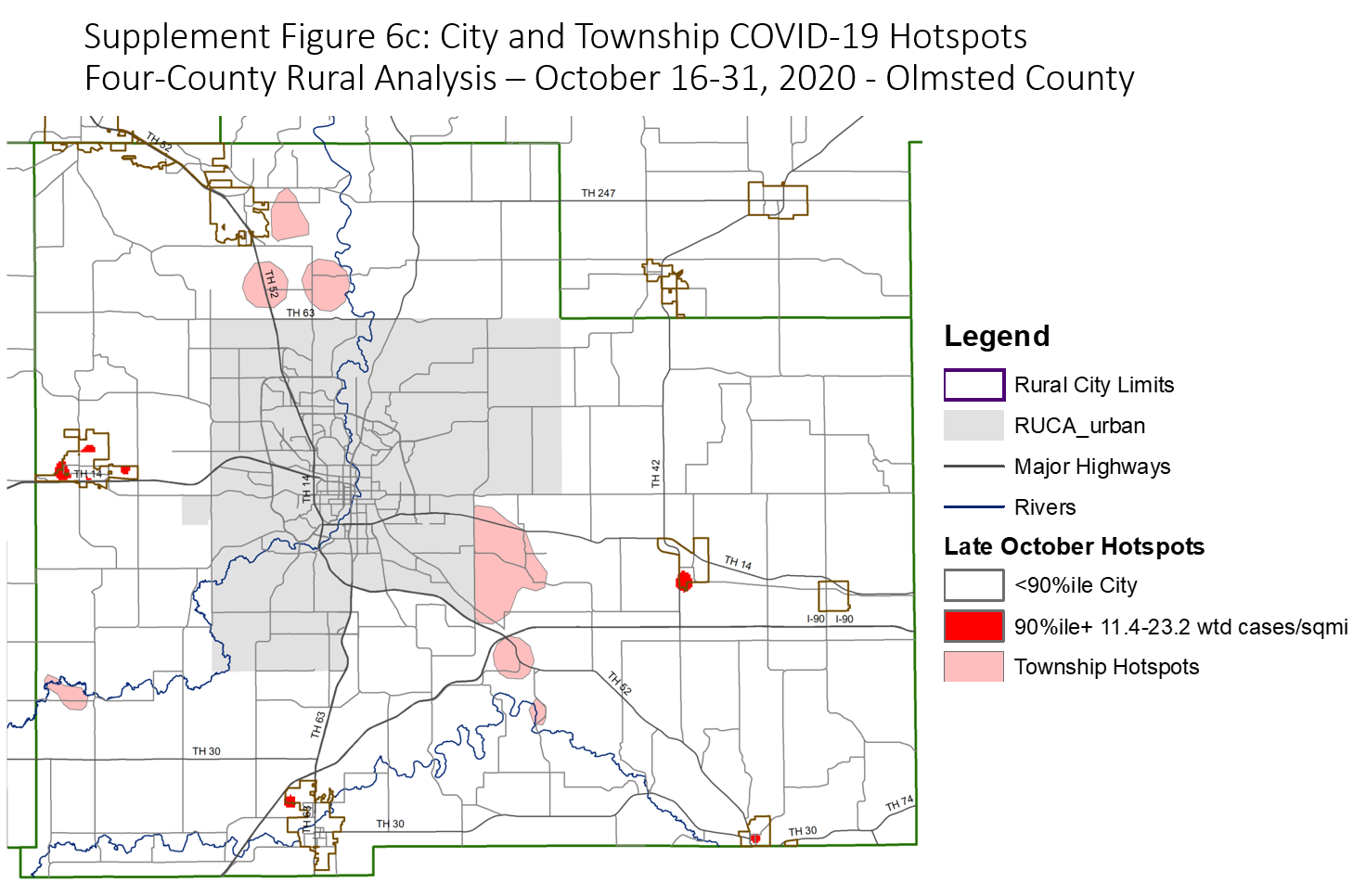

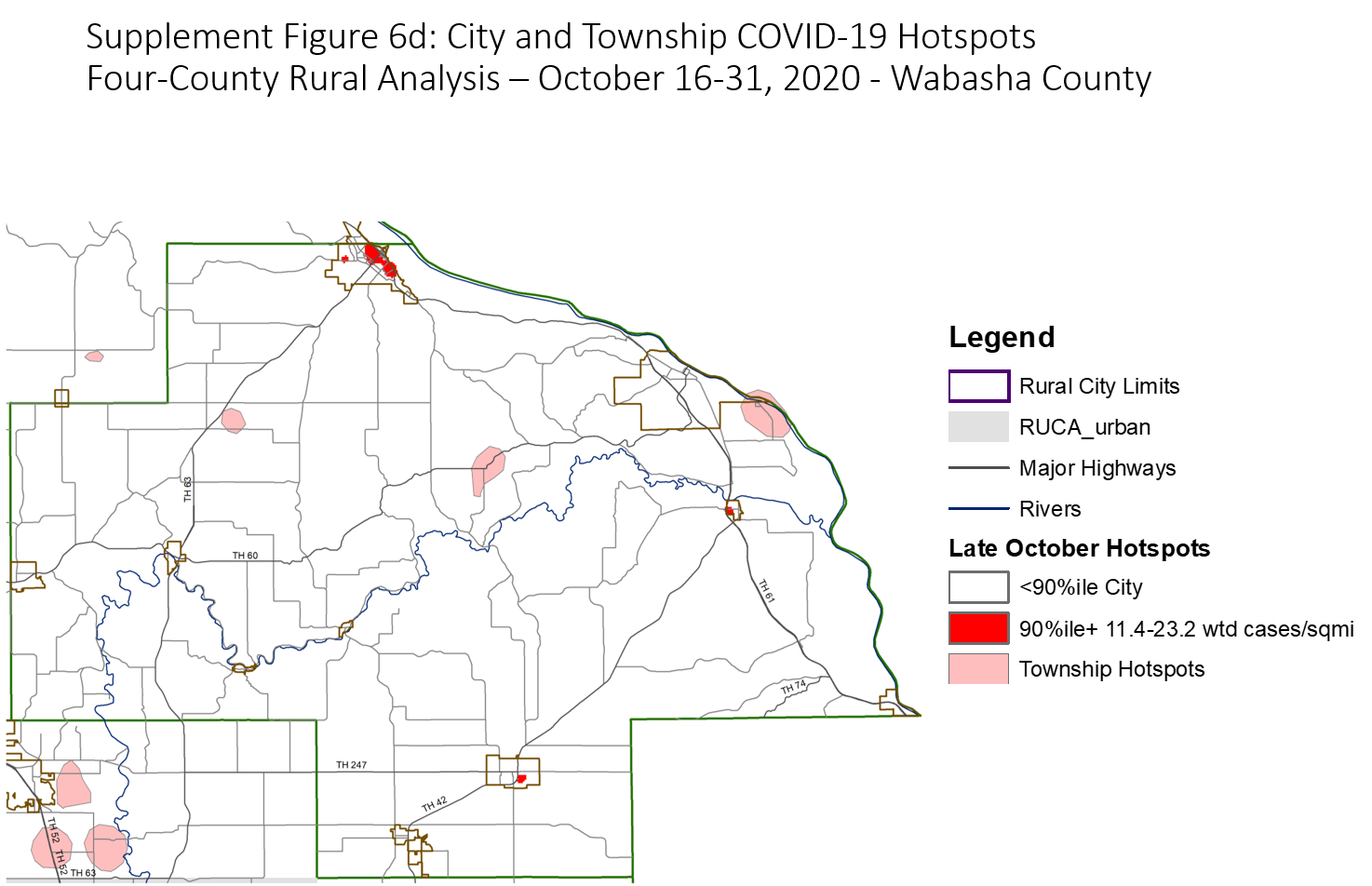

Supplement: Supplementary file 1 [file S2059866121008852sup001.docx]
